# Supplementary material for: Using an objective computer task (QbTest) to aid the identification of attention deficit hyperactivity disorder (ADHD) in the Children and Young People Secure Estate (CYPSE): a feasibility randomised controlled trial
Source: BMJ Open. 2022 Dec 16;12(12):e064951. doi: 10.1136/bmjopen-2022-064951 (PMC9764651; doi:10.1136/bmjopen-2022-064951)
Supplement: Author's manuscript [file bmjopen-2022-064951.draft_revisions.pdf]

BMJ Open

BMJ Open is committed to open peer review. As part of this commitment we make the peer review history of every article we publish publicly available.

When an article is published we post the peer reviewers' comments and the authors' responses online. We also post the versions of the paper that were used during peer review. These are the versions that the peer review comments apply to.

The versions of the paper that follow are the versions that were submitted during the peer review process. They are not the versions of record or the final published versions. They should not be cited or distributed as the published version of this manuscript.

BMJ Open is an open access journal and the full, final, typeset and author-corrected version of record of the manuscript is available on our site with no access controls, subscription charges or pay-per-view fees (http://bmjopen.bmj.com).

If you have any questions on BMJ Open's open peer review process please email info.bmjopen@bmj.com

BMJ Open

Using an objective computer task (QbTest) to aid the identification of Attention Deficit Hyperactivity Disorder (ADHD) in the Children and Young People Secure Estate (CYPSE): A feasibility randomised controlled trial

Journal:	*BMJ Open*
Manuscript ID	bmjopen-2022-064951
Article Type:	Original research
Date Submitted by the Author:	22-May-2022
Complete List of Authors:	Chitsabesan, Prathiba; Pennine Care NHS Foundation Trust, Children and Young People's Research Unit<br>Hall, Charlotte; Institute of Mental Health, NIHR CLAHRC-East Midlands, Psychiatry<br>Carter, Lesley-Anne; The University of Manchester, Centre for Biostatistics, Division of Population Health, Health Services Research and Primary Care<br>Reeves, Mindy; The University of Manchester<br>Mohammed, Vaseem; The University of Manchester<br>Beresford, Bryony; University of York, Social Policy Research Unit<br>Young, Susan; University of Reykjavik,<br>Kraam, Abdullah ; Rotherham Doncaster and South Humber Mental Health NHS Foundation Trust, Children and Adolecent Mental Health<br>Trowse, Sally; Pennine Care NHS Foundation Trust<br>Wilkinson-Cunningham, Lloyd; Leeds Community Healthcare NHS Trust,<br>Lennox, Charlotte; University of Manchester, Manchester Academic Health Science Centre
Keywords:	MENTAL HEALTH, Developmental neurology & neurodisability < PAEDIATRICS, Child & adolescent psychiatry < PSYCHIATRY

SCHOLARONE™
Manuscripts

I, the Submitting Author has the right to grant and does grant on behalf of all authors of the Work (as defined in the below author licence), an exclusive licence and/or a non-exclusive licence for contributions from authors who are: i) UK Crown employees; ii) where BMJ has agreed a CC-BY licence shall apply, and/or iii) in accordance with the terms applicable for US Federal Government officers or employees acting as part of their official duties; on a worldwide, perpetual, irrevocable, royalty-free basis to BMJ Publishing Group Ltd ("BMJ") its licensees and where the relevant Journal is co-owned by BMJ to the co-owners of the Journal, to publish the Work in this journal and any other BMJ products and to exploit all rights, as set out in our licence.

The Submitting Author accepts and understands that any supply made under these terms is made by BMJ to the Submitting Author unless you are acting as an employee on behalf of your employer or a postgraduate student of an affiliated institution which is paying any applicable article publishing charge ("APC") for Open Access articles. Where the Submitting Author wishes to make the Work available on an Open Access basis (and intends to pay the relevant APC), the terms of reuse of such Open Access shall be governed by a Creative Commons licence – details of these licences and which Creative Commons licence will apply to this Work are set out in our licence referred to above.

Other than as permitted in any relevant BMJ Author's Self Archiving Policies, I confirm this Work has not been accepted for publication elsewhere, is not being considered for publication elsewhere and does not duplicate material already published. I confirm all authors consent to publication of this Work and authorise the granting of this licence.

For peer review only

Using an objective computer task (QbTest) to aid the identification of Attention Deficit Hyperactivity Disorder (ADHD) in the Children and Young People Secure Estate (CYPSE): A feasibility randomised controlled trial

Authors and affiliations

1. Prathiba Chitsabesan[1,2],
2. Charlotte Lucy Hall[3],
3. Lesley-Anne Carter[4],
4. Mindy Reeves[5],
5. Vaseem Mohammed[5],
6. Bryony Beresford[6],
7. Susan Young[7],
8. Abdullah Kraam[8],
9. Sally Trwose[1],
10. Lloyd Wilkinson-Cunningham[9],
11. Charlotte Lennox[10]

Corresponding author- Prathiba Chitsabesan - pchitsabesan@nhs.net

1. Child and Adolescent Mental Health Service, Pennine Care NHS Foundation Trust, Ashton-under-Lyne, Lancashire, UK.

2. Division of Health Psychology and Social Care, Manchester Metropolitan University, Manchester, UK

3. NIHR MindTech MedTech Cooperative, Mental Health & Clinical Neurosciences, Institute of Mental Health, University of Nottingham, Nottingham, Nottinghamshire, UK

4. Centre for Biostatistics, Division of Population Health, Health Services Research and Primary Care, The University of Manchester, Manchester, UK

5. Medical School, The University of Manchester, Manchester, UK

6. Social Policy Research Unit, University of York, York, North Yorkshire, UK

7. Department of Clinical and Forensic Psychology, Psychology Services Limited, London, UK and Department of Psychology, Reykjavik University, Iceland

For peer review only - http://bmjopen.bmj.com/site/about/guidelines.xhtml

8. Child and Adolescent Mental Health Service, Rotherham Doncaster and South Humber Mental Health NHS Foundation Trust, Doncaster, UK

9. Research and Development, Leeds Community Healthcare NHS Trust, Leeds, UK

10. Faculty of Biology, Medicine and Health, School of Health, Division of Psychology and Mental Health, The University of Manchester, Manchester, UK

Abstract

Objectives: QbTest has been shown to improve time to decision/diagnosis for young people with Attention Deficit Hyperactivity Disorder (ADHD). The aim was to assess feasibility of QbTest for young people in prison.

Design: Single centre feasibility Randomised Controlled Trial (RCT), with 1:1 allocation. Concealed random allocation using an online pseudo-random list with random permuted blocks of varying sizes.

Setting: One Young Offenders Institution (YOI) in England.

Participants: 355 young people aged 15-18 years displaying possible symptoms of ADHD were assessed for eligibility, 69 were eligible to take part and 60 were randomised.

Intervention: QbTest - a computer task measuring attention, activity, and impulsivity.

Main outcome measures: Eligibility, recruitment and retention rates and acceptability of randomisation and trial participation.

Results: Of the 355 young people assessed for eligibility, 69 were eligible, and 60 were randomised (n = 30 QbTest plus usual care; n = 30 usual care alone). The study achieved the specified recruitment target. Trial participation and randomisation were deemed acceptable by the majority of participants. 78% of young people were followed up at 3-months, but only 32% at 6-months, although this was also affected by COVID restrictions. Secondary outcomes were mixed. Participants including clinical staff were mostly supportive of the study and QbTest, however some young people found QbTest hard and there were issues with implementation of the ADHD care pathway. There were no serious adverse events secondary to the study or intervention and no one was withdrawn from the study due to an adverse event.

Conclusions: With adaptations, a fully powered RCT may be achievable to evaluate the effectiveness of QbTest in the assessment of ADHD in the CYPSE, with time to decision (days) as the primary outcome measure. However, further programme developmental work is required to address some of the challenges highlighted prior to a larger trial.

For peer review only - http://bmjopen.bmj.com/site/about/guidelines.xhtml

Trial registration: ISRCTN17402196

Funding: NIHR RfPB (Grant number: PB-PG-1216-20007)

Strengths and limitations

- The study has contributed to developing the evidence base in understanding barriers and facilitators to implementing QbTest within a secure estate setting.
- The study produced important feasibility information on eligibility, recruitment, attrition rates and collection of primary outcomes.
- The sample was obtained from only one site within the secure estate and includes boys only.
- While this was a pragmatic trial to assess feasibility, there were challenges identified in the usual care pathway and the number of diagnostic decisions within the follow up period was limited.
- COVID restrictions had an impact on delivery of usual care, access to the prison and collection of some of the outcome measures.

For peer review only

Background

Attention Deficit Hyperactivity Disorder (ADHD) is a common mental health disorder; signficanly greater in prevalence in the Children and Young People Secure Estate (CYPSE) up to 30.1% [1]; than the general population of young people 5% [2]. However, a recent report raised significant concerns about the number of young people in the CYPSE with undetected neurodisability, including ADHD, due to a lack of appropriate screening and assessment processes and training of staff [3].

A clinical assessment of ADHD requires integration of information including observation and reports from parents, teachers, and young people. This can be difficult to obtain for young people in the CYPSE. This approach is also reliant on subjective measures which can lead to a lack of reliability and consistency in the diagnosis of ADHD [4] or delays when there is a difference of opinion between informants [5]. All these factors can lead to delays in receiving a diagnosis and accessing evidence-based treatment. Early diagnosis and timely interventions reduce the risk of adverse long-term outcomes that are associated with ADHD such as antisocial behaviour, poor academic performance, and social functioning [6]. The social and economic burden of untreated ADHD on society is significant [7-9] and is estimated to be around £100,000 per case [10], although this does not include any criminal justice costs. There are effective treatments for ADHD including medication that can improve outcomes for young people [11,12]. One way of supporting the assessment for ADHD is the use of objective measures.

One objective measure is the continuous performance test (CPT). There several variants of the but all measure vigilance and sustained attention. While CPT demonstrates good sensitivity to ADHD and correlates well with symptoms [13], studies have shown significant overlap in the performance of children with ADHD and typically developing children [14] and variability in intellectual ability may confound the interpretation of CPT performance in ADHD [15].

However, recent evidence suggests that combining a CPT with an objective measure of motor activity may add value in the clinical assessment of ADHD [16-17].

QbTest (Qbtech Ltd) combines a computerised CPT with an infrared camera to detect motor activity during the test and provides an objective standardised measurement of attention, impulsivity and activity,. QbTest is highly correlated with blinded observer ratings of ADHD symptoms in placebo-controlled trials [18] and can help differentiate ADHD from other conditions [19].

For peer review only - http://bmjopen.bmj.com/site/about/guidelines.xhtml

In studies designed to assess ‘stand-alone’ diagnostic accuracy, QbTest has only moderate sensitivity and low specificity to ADHD [20]. However, these studies used QbTest independently of other clinical information. QbTest is not designed to act as a ‘stand-alone’ tool; the US FDA approved QbTest as a decision-aid tool to augment, but not replace, standard clinical assessment of ADHD. This assessment approach has been shown to be acceptable to both families and clinicians [21].

A recently published single blind randomised control trial [22] demonstrated positive results for the role of Qbtest in supporting ADHD diagnosis alongside a routine ADHD assessment. At 6 months, clinicians with access to the QbTest report were more likely to reach a diagnostic decision about ADHD, felt more confident in their decision and took less consultation minutes to reach the decision compared to those who had not seen a QbTest report. There was no difference in diagnostic accuracy. The authors concluded QbTest may increase the efficiency of ADHD assessment pathway allowing greater patient throughput with clinicians reaching diagnostic decisions faster without compromising diagnostic accuracy.

Despite research evidence demonstrating service efficiencies to community clinical services alongside feasibility and acceptability, [16-17, 19, 22-23] there are several unknowns in relation to its use in the CYPSE including adjustments for the prison regime and complexity of the young people and therefore we undertook a feasibility study.

Aims and objectives

To assess the feasibility and acceptability of conducting a pragmatic RCT of QbTest in the assessment of ADHD in the CYPSE.

Methods

Trial Design

We conducted a single centre parallel two-group feasibility RCT with 1:1 individual participant allocation to QbTest plus usual care or usual care alone. Recruitment started 26March 2019 and ended 27 February 2020. Six-month follow-up continued until October 2020. The primary aim of the study was to assess the feasibility and acceptability of conducting a pragmatic RCT of QbTest in the assessment of ADHD in the CYPSE. We were interested to evaluate whether outcomes measures could be reliably collected at baseline and follow up and to help identify primary and secondary measures for a larger RCT.

For peer review only - http://bmjopen.bmj.com/site/about/guidelines.xhtml

Participants

The study took place in one Young Offenders Institution (YOI) accommodating boys aged 15-18 years in England. Participants were eligible if they answered a 'yes' to any of the ADHD symptoms on the CHAT assessment (Comprehensive Health Assessment Tool[1]). Participants were excluded if: they were on remand, did not speak English, had a previous or current diagnosis of ADHD, deemed a risk to either the researcher or staff, unable to provide informed consent (over 16 years) or parent/legal guardian consent was not received (under 16). The flow of participants is shown in figure 1. A sample size of 60 (30 to each group) were large enough to test the feasibility of the research procedures [24].

Insert figure 1 about here

Usual Care

Within the YOI, the expected usual care was as follows: young people identified with potential ADHD are offered an initial appointment by the neurodevelopmental lead, and initially assessed to decide whether the young person potentially has ADHD. This appointment was expected to be offered within a month of randomisation. If there were potential ADHD symptoms then an Assistant Mental Health Practitioner would commence the assessment by completing questionnaires, collecting a developmental history, and observing the young person. The time frame for this varies, but would aim to be completed within two months of randomisation. Once the history, observation and informant information are collected, another appointment would be made for the young person with the neurodevelopmental lead to review the information and either diagnose with ADHD or exclude diagnosis. If a diagnosis were made, then medication would be trialled, and the young person monitored for any improvements in presentation or side effects from medication.

Intervention – Usual care plus QbTest

Prior to the triage appointment by the neurodevelopmental lead, young people in the QbTest group were booked into the QbTest clinic. QbTest (Qbtech Ltd) is a computer task that

[1] The CHAT is a health needs assessment used in the CYPSE at the point of admission. It assesses first night risk e.g. self-harm, followed by a comprehensive assessment of physical and mental health, substance use and neurodisability over the first 10 days of admission. The ADHD section on the CHAT contains symptom questions around concentration, restlessness/fidgeting, interrupting and difficult waiting turn.

For peer review only - http://bmjopen.bmj.com/site/about/guidelines.xhtml

measures three core aspects of ADHD: attention, impulsivity, and motor activity. Performance on the task provides information (via an immediate report) on the three symptom domains of ADHD and a 'summary score' based on deviation from a normative data set (for age and gender). The healthcare team conducted the QbTest and used the information from the QbTest report in conjunction with the clinical information to inform their decision whether the young person had ADHD or not.

Randomisation

Randomisation was completed by the research assistant and achieved by means of concealed random allocation using an online pseudo-random list hosted by Sealedenvelope.com with random permuted blocks of varying sizes.

Blinding

Given the nature of QbTest, it was not possible to blind participants or those involved in delivering the intervention. Participants and research team were blind to allocation at the time of the baseline measures being completed which occurred before randomisation. It was not possible to maintain the field researcher being blind to trial arm allocation because the researcher and staff delivering the intervention were both working within the confines of the YOI. Only the statistician (LAC) who was assessing the outcomes was blind to trial arm allocation.

Primary Outcomes

The following outcome measures and data were collected:

- Eligibility rate recorded as the number of eligible young people against the total number of young people assessed for eligibility (i.e., identified with ADHD needs on the CHAT);
- Recruitment rate recorded as the number of young people who consent to participate against the number of eligible young people;
- Acceptability of randomisation recorded as the number of young people randomised;
- Acceptability of trial participation recorded as the number of eligible young people who dropped out after receiving allocation;
- Retention rate recorded as the number of young people who were randomised that remain in the study until the end of follow up at 6 months (target of 50% at 6 months in both trial arms).

For peer review only - http://bmjopen.bmj.com/site/about/guidelines.xhtml

For peer review only

Secondary Outcomes

The secondary outcome measures selected included measures of ADHD symptoms, quality of life and service use. Other key data collected such as the number of appointments and days until diagnosis and number of behavioural episodes were also key outcome data that would be relevant to a larger study in measuring the impact of the intervention arm (QbTest) and identifying differences between the two groups (usual care vs usual care and QbTest group). The following outcome measures and data were collected at baseline, 3 and 6 months, unless otherwise stated:

Completed by the young person with support from the research assistant

- Behaviour measured using the Strengths and Difficulties Questionnaire (SDQ) [25]. The SDQ is a well validated and commonly used brief screening questionnaire of children's emotions and behaviours through 5 scales (e.g., emotional; conduct problems; hyperactivity/inattention; peer relationship problems and prosocial) scored 0-10. Scores are categorised from 'close to average' (80% of population) to 'very high' (5% of population).
- ADHD symptoms were measured using the Brief Barkley ADHD Rating Scale (B-BAARS) [26]. The Brief-Barkley Adult ADHD Rating Scale (B-BAARS) has been developed as a brief assessment to screen for ADHD in adults in the criminal justice system and has been shown to accurately predict the likelihood of receiving a clinical diagnosis using DSM-V criteria.
- Health-related quality of life was measured using the Child Health Utility Instrument (CHU-9D) [27] [2]. The CHU-9D is a quality of life measure containing 9 main dimensions (worried, sad, pain, tired, annoyed, schoolwork/homework, sleep, daily routine, and activities), each with 5 increasing levels of severity/impairment.
- Contact with services was measured using the modified Client Service Receipt Inventory (CSRI) [28]. It's allows resource use patterns to be described and support costs to be estimated using an appropriate unit cost.
- .At 3 months feedback from young people receiving the QbTest using the QbTest opinion questionnaire.

Completed by the clinical team

- The Children's Global Assessment Scale (C-GAS) [29] is a rating of functioning for young people 6-17years of age. The young person is given a single score between 1 and 100 based on a clinician's assessment of the young person's psychological and

[2] Only reported as level of missing data.

For peer review only - http://bmjopen.bmj.com/site/about/guidelines.xhtml

social functioning. The score puts them in one of ten categories that range from ‘extremely impaired’ (1-10) to ‘doing very well’ (91-100).

- Number of appointments until confirmed ADHD diagnosis/exclusion of ADHD and number of days until a decision was reached was recorded on a pro-forma completed by clinicians after each appointment with the young person.

Completed by the teacher

- The Swanson, Nolan, and Pelham Rating Scale (SNAP-IV) is a widely used scale that measures the core symptoms of attention deficit hyperactivity disorder (ADHD) [30]. Teachers are asked to rate 26 items on a 4point scale.

Completed by the research assistant from health and prison records

- Number of recorded behavioural incidents were collected using prison information.
- Numbers of young people agreeing to sit the QbTest and the number who completed the QbTest was recorded.

Qualitative interviews with young people and staff

- At 3 months participants were interviewed using a semi-structured interview schedule about the acceptability of randomisation, acceptability of outcome measures and for those in the QbTest group acceptability of the QbTest. Purposive sampling was used to select participants for interview including consideration of age, completion of the QbTest and scores on the QbTest opinion questionnaire.
- At the end of the study health professionals and CYPSE staff were interviewed on the acceptability and feasibility of administering and implementing QbTest within usual assessment practices as well as facilitators and barriers to using the QbTest and reasons for non-completion.

See study protocol [31] for details of how data collection was conducted.

Data Analysis

As a feasibility study, data analysis was mainly descriptive [32]. All measures were summarised by group across follow-up time with mean and standard deviation for normally

For peer review only - http://bmjopen.bmj.com/site/about/guidelines.xhtml

distributed data, median for skewed variables and frequency (percentage) for categorical data. All statistical analysis were conducted using Stata v.15.

Interviews were conducted and recorded by the researcher using an encrypted Dictaphone. All qualitative data were analysed using thematic analysis [33] with the aid of NVivo. Data were coded inductively into themes, creating a detailed coding scheme, allowing for the investigation of emergent patterns between individual codes and boarder emergent themes.

Patient and Public Involvement (PPI)

PPI was embedded in many aspects of the study including; the development of the funding application and shaping the study objectives and outcome measures. The burden of the intervention for young people had been previously assessed through a local community audit. Within the project we supported the development of a PPI group within the YOI and they met on a quarterly basis, facilitated by the research assistant. This group helped develop our Patient Information Sheet and advised on recruitment and data collection including the need for the researcher to be flexible in their approach eg seeing young people on the unit rather than the young people attending healthcare. COVID significantly impacted our ability to continue this group and as a result, our PPI group were not involved in the analysis or dissemination stages of the project as we had planned. However, our PPI representatives (two parents of young people with ADHD) have helped us interpret the qualitative data and write lay summaries and infographics for staff and young people. This will be disseminated to staff through a webinar and written summary and to the young people through the healthcare team.

Results

A total of 60 young people were randomised 30 to QbTest plus usual care (intervention) and 30 to usual care (control). Table 1 shows the demographics of the 60 boys randomised to the study. Of the 60 young people a sub-sample of 11 young people (6 QbTest v 5 usual care) were interviewed. The research assistant and five members of staff were also interviewed, including all those who were delivering QbTest and staff involved in supporting the implementation.

Table 1: Baseline demographic information

	QbTest (n = 30)		Usual Care (n = 30)	
	n	%	n	%

For peer review only - http://bmjopen.bmj.com/site/about/guidelines.xhtml

**Age**					
	**16**	6	20.0	3	10
	**17**	8	26.7	11	36.7
	**18**	15	50.0	16	53.3
	**Missing**	1	3.3	0	0
**Ethnicity**					
	**White**	23	76.7	24	80.0
	**Other**	6	20.0	6	20.0
	**Missing**	1	3.3	0	0
**Accommodation**					
	**House or flat**	20	66.7	25	83.3
	**Other**	9	30.0	5	16.7
	**Missing**	1	3.3	0	0
**Lived with**					
	**Parent(s)**	15	50.0	21	70.0
	**Other**	13	43.3	9	30.0
	**Missing**	2	6.7	0	0
**Education**					
	**Mainstream**	6	20.0	6	20.0
	**Pupil Referral Unit**	3	10.0	5	16.7
	**None**	20	66.7	17	56.7
	**Other**	0	0	2	6.7
	**Missing**	1	3.3	0	0

Primary Outcomes

Eligibility rate

Participant flow (see Figure 1) showed that over 12 months we assessed 355 young people for eligibility. The numbers and reasons for ineligibility were: 69 (19%) on remand, 109 (30%) had a previous diagnosis of ADHD, 94 (26%) were released or transferred before they could be approached, 6 (2%) were deemed too high risk, 4 (1%) were not fluent in English as a language and 4 (1%) were deemed to lack capacity. This gave us an eligibility rate of 19.4% (69/355).

Recruitment rate

Of the 69 young people who were eligible, 60 (87%) consented to participate in the study. For the nine participants who did not consent, their reason was either they did not feel they had ADHD or that they were leaving prison soon. Our initial estimates were to achieve an average recruitment rate of five randomisations a month (60 young people over 12 months) which was successfully achieved.

Acceptability of randomisation and trial participation

For peer review only - http://bmjopen.bmj.com/site/about/guidelines.xhtml

For peer review only

Of the 60 young people who consented to take part all agreed to be randomised and no young people refused to continue in the study after randomisation (see Figure 1).

Retention rate

Of the 60 young people 47 were followed up at 3-months, a retention rate of 78% which exceeded our target. Our aim was to successfully follow up at least 50% at 6 months. However, we were only able to follow up 19 of the 60 young people; a retention rate of 32%. Half of the 6-month follow-ups were due after March 2020 and therefore were significantly impacted by COVID as we were not able to access the YOI from March 2020 for 8months.

There were no serious adverse events secondary to the study or intervention and no one was withdrawn from the study due to an adverse event.

Secondary Outcomes

Of the 30 young people randomised to receive QbTest all agreed to sit the QbTest. At the end of the recruitment window four young people could not undertake their QbTest due to the COVID lockdown in the prison. For the remaining 26 young people, a total of 6 (23%) did not complete their QbTest: for three young people (12%) the appointment was not attended due to prison resources (staffing to accompany the young person), two (8%) young people were released/transferred before the QbTest could take place and one young person (4%) was unable to sit the QbTest due to concerns from the clinical team regarding increased risk of self-harm at the time. The median number of days between randomisation and QbTest was 42 (IQR = 26 – 93; min = 1; max = 195).

Table 2 shows the descriptive statistics for the questionnaires collected at baseline, 3 and 6 months for the two trial arms from the young people.

For peer review only

For peer review only - http://bmjopen.bmj.com/site/about/guidelines.xhtml

Table 2: Descriptive statistics for secondary outcome measures

		Baseline (n=60)				3 months (n=47)				6 months (n=19)			
		QbTest		Usual Care		QbTest		Usual Care		QbTest		Usual Care	
		N	%	n	%	n	%	n	%	n	%	n	%
**SDQ Total cut-off**													
	Close to Average	7	23.3	5	16.7	2	6.7	4	13.3	2	6.7	3	10.0
	Slightly Raised	4	13.3	8	26.7	0	0	5	16.7	0	0	4	13.3
	High	2	6.7	5	16.7	4	13.3	1	3.3	0	0	1	3.3
	Very High	16	53.3	12	40.0	7	23.3	7	23.3	7	23.3	1	3.3
	Missing	1	3.3	0	0	17	56.7	13	43.3	21	70.0	21	70.0
**B-BAARS**													
Likelihood of receiving	Yes	21	70.0	23	76.7	10	33.3	12	40.0	7	23.3	4	13.3
a clinical Diagnosis	No	8	26.7	7	23.3	3	10.0	6	20.0	2	6.7	4	13.3
(DSM V criteria)	Missing	1	3.3	0	0	17	56.7	12	40.0	21	70.0	22	73.3
**SNAP[a]**													
	Missing	26	86.7	27	90	30	100	30	100	30	100	30	100
**CHU-9D[a]**													
	Missing	2	6.7	0	0	17	56.7	13	43.3	21	70.0	22	73.3
**C-GAS[b]**													
	Some noticeable problems	3	10.0	0	0	0	0	0	0	0	0	0	0
	Some problems	3	10.0	0	0	1	3.3	0	0	0	0	0	0
	Doing all right	1	3.3	1	3.3	0	0	2	6.7	0	0	1	3.3
	Doing well	0	7	2	6.7	0	0	2	6.7	0	0	0	0
	Missing	23	76.7	27	90.0	29	96.7	26	86.7	30	100	29	96.7
**Service Use (CSRI)**													
Emergency	Yes	4	13.3	8	26.7	2	6.7	1	3.3	2	6.7	1	3.3
Attendance	No	23	76.7	22	73.3	11	36.7	16	53.3	7	23.3	7	23.3
Physical Health	Missing	3	10.0	0	0	17	56.7	13	43.3	21	70.0	22.0	73.3
Planned	*Yes*	1	3.3	0	0	0	0	0	0	0	0	0	0
Admission	*No*	26	86.7	30	100	13	43.3	17	56.7	9	30.0	8	26.7
Physical Health	*Missing*	3	10.0	0	0	17	56.7	13	43.3	21	70.0	22	73.3

For peer review only - http://bmjopen.bmj.com/site/about/guidelines.xhtml

Outpatient	Yes	2	6.7	2	6.7	1	3.3	4	13.3	0	0	1	3.3
Appointments	No	25	83.3	28	93.3	12	40.0	13	43.3	9	30.0	7	23.3
Physical Health	Missing	3	10.0	0	0	17	56.7	13	43.3	21	70.0	22	73.3
Missed	Yes	6	20.0	5	16.7	3	10.0	1	3.3	1	3.3	0	0
Appointments	No	21	70.0	25	83.3	10	33.3	16	53.3	8	26.7	8	26.7
	Missing	3	10.0	0	0	17	56.7	13	43.3	21	70	22	73.3
Emergency	Yes	1	3.3	2	6.7	0	0	0	0	0	0	0	0
Attendance	No	26	86.7	28	93.3	13	43.3	17	56.7	9	30.0	8	26.7
Menta Health	Missing	3	10.0	0	0	17	56.7	13	43.3	21	70.0	22	73.3
Planned	Yes	0	0	0	0	0	0	0	0	0	0	0	0
Admission Mental	No	24	80.0	30	100.0	13	43.3	16	53.3	9	30.0	8	26.7
Health	Missing	6	20.0	0	0	17	56.7	14	46.7	21	70.0	22	73.3
Missed	Yes	1	3.3	1	3.3	1	3.3	2	6.7	0	0	0	0
Appointments	No	25	83.3	29	96.7	12	40.0	15	50	9	30.0	7	23.3
Mental Health	Missing	4	13.3	0	0	17	56.7	13	43.3	21	70.0	23	76.7
**Number of Service Contacts (CSRI)**													
Physical Health	Median	3		2		4		1		2		1	
Contacts	IQR	1-8		1-4		1-8		0-6		2-3		0-12	
	Min Max	0, 47		0, 73		0, 88		0, 14		0, 6		0, 51	
	n	27		30		13		17		9		8	
Mental Health	Median	6		4		2		5		2		0.5	
Contacts	IQR	2-20		1-6		2-4		4-12		1-5		0-1.5	
	Min Max	0, 89		0,333		0, 18		0, 24		0,37		0,5	
	n	27				13		17		9		8	
Criminal Justice	Median	9		8.5		4		4		2		5	
Contacts	IQR	3-23		5-20		3-6		3-6		1-4		2.5-26	
	Min Max	1,107		1,106		0,65		0,16		0,137		2,60	
	n	27		30		13		17				8	
**Number of Incidents**													
	Median	1		1		2		2		3.5		7	
	IQR	0-2		0-2		1-5		0-6.5		1-4		2-7	
	Min Max	0, 12		0, 19		0, 12		0, 19		1, 7		0, 8	
	n	29		29		9		12		6		5	

a -missing data presented only

b- CGAS -has 10 categories from extremely impaired to doing very well- relevant categories presented only

For peer review only

For peer review only - http://bmjopen.bmj.com/site/about/guidelines.xhtml

The amount of missing data for all outcomes are shown in Table 2. Of those contactable for the 3 month follow up, 64%-66% completed the SDQ, B-BAARRS and CHU-9D. Completion rates of the 6 month measures were higher, however only 19 participants were contactable. The C-GAS and SNAP were poorly completed across all time points. As the study aimed to assess the feasibility of collecting outcome measures, we have not made comparisons of the data between intervention arms and over time points.

Of the 60 young people, a total of 32 (53%) received at one appointment and three (5%) had two appointments. Therefore, a total of 25 young people (42%) did not receive any appointment within the 6-month follow-up window. Some young people did have appointments, which fell outside of the 6-month window. Appointments had been offered to some young people but were not attended (n = 53).

The low number of appointments offered and attended within the 6month follow up period impacted on the number of decisions being made. We recorded evidence of 14 decisions across the 60 participants, 8 in the QbTest group and 6 in the usual care group (27% event rate vs 20%). All these decisions were exclusions of ADHD; there were no diagnoses of ADHD for any young people.

Data from the qualitative interviews suggested that most of the young people had some understanding of the randomisation process and were able to explain what had happened. Most of the young people said that they were happy or were not upset about being allocated to either group. There were however two young people who expressed stronger feelings about the group to which they were allocated. One was angry about being allocated to the QbTest group because they did not like the test as it took too long, and the other was allocated to usual care but wanted to receive QbTest as they thought they had ADHD. The young people were all happy with being involved in the research and one young person said that they would be happy to recommend the study to others.

Of the 20 young people who had received QbTest, 10 completed the QbTest questionnaire. Table 3 shows an overview of the data.

Table 3: Descriptive statistics for the QbTest feedback questionnaire

Item	Disagree	Neither	Agree
The QbTest results helped me understand my symptoms	2	6	2
The QbTest results were difficult to understand	1	7	2
I fully understood the purpose of the QbTest	1	0	9
I found the assessment very stressful	1	0	9

For peer review only - http://bmjopen.bmj.com/site/about/guidelines.xhtml

The task took too long to complete	0	1	9
I found the task difficult to complete	1	1	8
Overall the experience was useful	3	5	2
I found the chair very uncomfortable	3	5	2
The QbTest helped me understand the changes in my symptoms since my previous appointment[a]	1	7	1
I found the QbTest much easier to complete when on meds[b]	**0**	**6**	**0**
When the clinician talked through the output with me, it helped me understand how s/he had reached their diagnosis[a]	**0**	**6**	**3**
When the clinician talked me through the output, it helped me understand how they had reached their decision about medication	0	6	2

a = missing data for one young person b = missing data for four young people;

The questionnaire feedback suggests that young people may not have seen a significant benefit from undertaking the QbTest although not all had received feedback on the test at the time they completed the QbTest questionnaire. Data from the qualitative interviews also suggested that while some young people had been fine at the beginning of the test, they had found the process long and at times boring or felt exhausted by the end. One young person had to repeat the test and admitted feeling cross by this although another said he would recommend QbTest to other young people. At the time of the interview, not all young people had received the outcome of the test and ADHD assessment. One young person reported feeling excited by the possibility of a diagnosis and happy that he would be receiving help. Another young person was told that he did not have ADHD after the QbTest assessment but was unhappy with this as he did not feel this was correct.

Feedback from staff was generally more positive although they identified some challenges in using QbTest within a YOI setting. Initially there had been some concerns because of the equipment (laptop and camera) and IT system needed but it was felt that it was easy to address. The main difficulties appeared to be finding an appropriate room for the equipment which was quiet for the testing environment. While it had been planned that all QbTests would be undertaken in the same room and the equipment set up; staff did have to move to other rooms on occasion. One member of staff said that it was easy to carry the equipment around and set up in another location if needed. There were problems with the prison staff facilitating the appointments in healthcare (e.g., challenges in bringing the young people over to healthcare for their appointment). The healthcare staff often had to offer multiple appointments for the young person to attend to take the QbTest.

The staff members interviewed were of the opinion that QbTest was helpful in assessing ADHD and one staff member also reported that it helped both the young person and staff understand the young person's behaviours better. Staff felt that the QbTest could be helpful

For peer review only - http://bmjopen.bmj.com/site/about/guidelines.xhtml

in improving waiting times and worked well alongside the ADHD clinical assessment. One staff member felt the QbTest was more objective and there was less bias than self-report measures. All the staff interviewed felt that the QbTest should be used in other sites as there were many advantages of using the QbTest. One staff member added that the QbTest was helpful in the assessment of complex young people, where there might be concerns about co-morbid diagnosis.

Discussion, Limitations and Lessons Learnt

Despite the impact of COVID restrictions, this study has provided important information regarding the feasibility of introducing QbTest into the secure estate and conducting a larger trial. Our primary outcomes were largely achieved; estimates of eligibility were broadly accurate, consent rates were high, we reached our recruitment target on time, randomisation and trial participation was high. We were able to follow-up young people, but this was easier over 3 months than 6 months and where the young people stayed within the YOI. Our secondary outcomes were more mixed; we were able to collect some outcome measures more easily than others although this was impacted by COVID restrictions. However, only 23% of young people received a decision on diagnosis while they were within the secure estate which were all exclusion of ADHD. This may reflect the complexity of the needs of young people in the secure estate and the challenges in undertaking assessments within this environment.

Participants (both young people and staff) were largely supportive of the study and the role of QbTest. We know from clinical practice, that young people can find QbTest challenging as it tests areas that they struggle with. This was evident from the questionnaire feedback and the interviews. However, this has also been reported in other studies and is experienced in clinical practice [21].

Similar to the AQUA trial [22] we allowed the clinical team to complete QbTest at a time that suited them. Although we had suggested that it would be ideal for the clinical team to undertake the QbTest within two weeks from randomisation the average number of days between randomisation and QbTest was much longer. Also, from the appointment data it suggested that not all young people were receiving usual care within the timeframes described by the clinical team at the start of the study and many young people did not receive any appointments. Young people not receiving appointments were across both trial arms and throughout recruitment, although it was exacerbated by the impact of COVID-19. Staffing shortages, appointments not being attended, short sentences and the rigidity of the prison regime were also contributing factors to fewer diagnostic decisions being made. Recently a

For peer review only - http://bmjopen.bmj.com/site/about/guidelines.xhtml

consensus group of UK experts highlighted the issues within current ADHD care pathways [34], given the complexity of the CYPSE it is likely that these issues are exacerbated.

Conclusion

Aspects of this feasibility trial demonstrated that a larger RCT trial of the effectiveness of QbTest within the CYPSE could be feasible, with time to decision (days) as the primary outcome measure . However, features of the intervention implementation were mixed, partly exacerbated by the impact of COVID. However, prior to progressing this additional programme developmental work would need to be undertaken. This would involve the review of the usual care ADHD pathway and the development of a standardised ADHD care pathway across the CYPSE as well as strategies to improve collection of some secondary outcome measures. This would aim to increase the number of appointments young people have with healthcare teams and subsequently decisions on ADHD status, enabling a better estimate of a time to decision for usual care time and the required sample size for a large multi-site RCT.

Contributors

PC: Chief Investigator, conceptualisation, supervision, methodology, writing and editing; CLH: Conceptualisation, methodology, writing – review and editing; LAC: Statistician, conceptualisation, methodology, analysis, writing – review and editing; BB: Conceptualisation, methodology, writing – review and editing; SY: Conceptualisation, methodology, writing – review and editing; AK: Conceptualisation, methodology, writing – review and editing; HL: PPI representative, analysis, writing – review and editing; ST: PPI representative, analysis, writing – review and editing; LWC: Researcher, data collection, writing – review and editing; MR: Researcher, data collection, data entry, analysis, writing – review and editing; VM: Researcher, data collection, data entry, analysis, writing – review and editing; CL: Project Manager, conceptualisation, methodology, data entry, analysis, writing and editing;

Acknowledgements

The active involvement of all young people and staff who participated within this research study and provided advice is gratefully acknowledged.

For peer review only - http://bmjopen.bmj.com/site/about/guidelines.xhtml

Disclaimer

The views expressed are those of the author(s) and not necessarily those of the NHS, the NIHR of the Department of Health. The study sponsor and funders have no role in study design, including collection, management, analysis and interpretation of data; writing of the report and the decision to submit the report for publication

Competing interests

The authors declare that they have no competing interests.

Funding

The study is funded by the NIHR RfPB (Grant number: PB-PG-1216-20007).

Availability of data and material

No additional data is available due to the population of research participants involved in this study.

Ethics Approval

All participants (or their parent or legal guardian) gave written informed consent to participate in the study. Ethical approval for the study was obtained from the Health and Research Care Wales; REC reference 18/WA/0347 and IRAS project ID 238947.

For peer review only

References

[1] Young S, Moss D, Sedgwick O, Fridman M, Hodgkins P. (2015) A meta-analysis of the prevalence of attention deficit hyperactivity disorder in incarcerated populations. Psychological Medicine, 45(2):247-58. doi: 10.1017/S0033291714000762.

[2] Fazel, S., Doll, H., & Langstrom, N. (2008). Mental disorders among adolescents in juvenile detention and correctional facilities: a systematic review and metaregression analysis of 25 surveys. Journal of the American Academy of Child and Adolescent Psychiatry 47, 1010–1019.

For peer review only - http://bmjopen.bmj.com/site/about/guidelines.xhtml

[3] Hughes, N., Williams, H., Chitsabeasan, P., Davies, R. & Mounce, L. (2012). Nobody made the connection: The prevalence of neurodisability in young people who offend. The Children's Commissioner https://www.childrenscommissioner.gov.uk/sites/default/files/publications/Nobody%20made%20the%20connection.pdf

[4] Ogundele, M.O., Ayyash, H.F. and Banerjee, S. (2011), Role of computerised continuous performance task tests in ADHD. Progress in Neurology and Psychiatry, 15: 8-13. https://doi.org/10.1002/pnp.198

[5] Kovshoff H, Williams S, Vrijens M, Danckaerts M, Thompson M, Yardley L, Hodgkins P, Sonuga-Barke EJ. The decisions regarding ADHD management (DRAMa) study: uncertainties and complexities in assessment, diagnosis and treatment, from the clinician's point of view. European Child & Adolescent Psychiatry. 2012;21(2):87–99

[6] Shaw, M., Hodgkins, P., Caci, H., Young, S., Kahle J., et al., (2012). A systematic review and analysis of long-term outcomes in attention deficit hyperactivity disorder: effects of treatment and non-treatment. *BMC Medicine* 10, 99

[7] Biederman, J., Petty, C., Clarke, A., Lomedico, A. & Faraone, S. V. (2010). Predictors of persistent ADHD: An 11-year follow-up study. Journal of Psychiatry Research 45, 150-155.

[8] Arnold, L. E., Hodgkins, P., Kahle, J., Madhoo, M., & Kewley, G. (2015). Long-term outcomes of ADHD: Academic achievement and performance. Journal of Attention Disorders, 1-13.

[9] D'Amico, F., Knapp, M., Beecham, J., Sandberg, S., Taylor, E. et al (2014). Use of services and associated costs for young adults with childhood hyperactivity/conduct problems: 20-year follow up. The British Journal of Psychiatry 204, 441-447.

[10] Khong ,B. (2014). The lifetime costs of attention deficit hyperactivity disorder (ADHD). Centre for Mental Health https://www.centreformentalhealth.org.uk/costs-of-adhd

[11] National Institute for Health and Care Excellence (2008). Attention deficit hyperactivity disorder: diagnosis and management (CG72). National Institute for Health and Care Excellence. https://www.nice.org.uk/guidance/cg72?unlid=520684933201611441814

[12] National Institute for Health and Care Excellence (2013). Attention deficit hyperactivity disorder (QS39). National Institute for Health and Care Excellence. https://www.nice.org.uk/guidance/qs39

For peer review only - http://bmjopen.bmj.com/site/about/guidelines.xhtml

[13] Epstein, J.N., Erkanli, A., Conners, C.K., Klaric, J., Costello, J.E., & Angold, A. (2003). Relations between continuous performance test performance measures and ADHD behaviors. Journal of Abnormal Child Psychology, 31, 543–554.

[14] Zelnik, N., Bennett-Back, O., Miari, W., Goez, H.R., & FattalValevski, A. (2012). Is the test of variables of attention reliable for the diagnosis of attention-deficit hyperactivity disorder (ADHD)? Journal of Child Neurology, 27, 703–707.

[15] Park, M.H., Kweon, Y.S., Lee, S.J., Park, E.J., Lee, C., & Lee, C.U. (2011). Differences in performance of ADHD children on a visual and auditory continuous performance test according to IQ. Psychiatry Investigation, 8, 227–233.

[16] Hall, C.L., Valentine, A.Z., Groom, M.J., Walker, G.M., Sayal, K., Daley, D., & Hollis, C. (2016). The clinical utility of the continuous performance test and objective measures of activity for diagnosing and monitoring ADHD in children: A © 2018 The Authors. Journal of Child Psychology and Psychiatry published by John Wiley & Sons Ltd on behalf of Association for Child and Adolescent Mental Health.

[17] Gilbert, H., Qin, L., Li, D., Zhang, X., & Johnstone, S.J. (2016). Aiding the diagnosis of AD/HD in childhood: Using actigraphy and a continuous performance test to objectively quantify symptoms. Research in Developmental Disabilities, 59, 35–42.

[18] Wehmeier, PM., Schacht, A., Wolff, C., Otto, WR., Dittmann, RW., & Banaschewski, T. (2011) Based Continuous Performance Test Combined with an Infra-Red Motion-tracking Device. *Journal of Child and Adolescent Psychopharmacology*, 21: 5, 433-444.

[19] Vogt, C., & Shameli, A. (2011). Assessments for attention-deficit hyperactivity disorder: use of objective measurements. The Psychiatrist 35, 380-383.

[20] Hult, N., Kadesjo, J., Kadesj € o, B., Gillberg, C., & Billstedt, E. € (2015). ADHD and the QbTest: Diagnostic validity of QbTest. Journal of Attention Disorders. https://doi.org/10.1177/1087054715595697

[21] Hall, C.L., Valentine, A.Z., Walker, G.M. et al. Study of user experience of an objective test (QbTest) to aid ADHD assessment and medication management: a multi-methods approach. BMC Psychiatry 17, 66 (2017). https://doi.org/10.1186/s12888-017-1222-5

[22] Hollis C, Hall CL, Guo B, James M, Boadu J, Groom MJ, Brown N, Kaylor-Hughes C, Moldavsky M, Valentine AZ, Walker GM, Daley D, Sayal K, Morriss R; the AQUA Trial Group. (2018) The impact of a computerised test of attention and activity (QbTest) on diagnostic decision-making in children and young people with suspected attention deficit hyperactivity

For peer review only - http://bmjopen.bmj.com/site/about/guidelines.xhtml

disorder: single-blind randomised controlled trial. Journal of Child Psychology and Psychiatry, 59(12):1298-1308.

[23] Hall, C., Selby, K., Boliang, G, Althea, V., Walker, G. & Hollis, C. (2016) Innovations in Practice: an objective measure of attention, impulsivity and activity reduces time to confirm attention deficit/hyperactivity disorder diagnosis in children – a completed audit cycle. Child and Adolescent Mental Health 21, 175–178.

[24] 31 Billingham, S. A, Whitehead, A. L., & Julious, S. A. (2013). An audit of sample sizes for pilot and feasibility trials being undertaken in the United Kingdom registered in the United Kingdom Clinical Research Network database. BMC Medical Research Methodology, 13, 104.

[25] Goodman, R. (1997). The Strengths and Difficulties Questionnaire: A research note. *Journal of Child Psychology and Psychiatry* 38, 581–586.

[26] Young, S., González, R. A., Mutch, L., Mallet-Lambert, I., O'Rourke, L. et al. (2011) Diagnostic accuracy of a brief screening tool for attention deficit hyperactivity disorder in UK prison inmates. Psychological Medicine 46, 1449-1458.

[27] Stevens K. Assessing the performance of a new generic measure of health-related quality of life for children and refining it for use in health state valuation. Applied Health Economics Health Policy. 2011;9:157–69.

[28] Beecham, J.& Knapp, M. (2001) Costing psychiatric interventions, in G. Thornicroft (ed.) Measuring Mental Health Needs, Gaskell, 2nd edition, 200-224.

[29] Swanson, J. M., Kraemer, H. C., Hinshaw, S. P., Arnold, L. E., Conners, C. K., et al (2001). Clinical relevance of the primary findings of the MTA: success rates based on severity of ADHD and ODD symptoms at the end of treatment. Journal of the American Academy of Child and Adolescent Psychiatry 40, 168-79.

[30] Shaffer, N., Gould, M. S., Brasic, J. (1983). A children's Global Assessment Scale (CGAS). Archives of General Psychiatry 40, 1228-1231.

[31] Lennox C, Hall CL, Carter L, et al FACT: a randomised controlled trial to assess the feasibility of QbTest in the assessment process of attention deficit hyperactivity disorder (ADHD) for young people in prison—a feasibility trial protocol BMJ Open 2020;10:e035519. doi: 10.1136/bmjopen-2019-035519

[32] Lancaster GA, Dodd S , Williamson PR . Design and analysis of pilot studies: recommendations for good practice. J Eval Clin Pract 2004;10:307–12. doi:10.1111/j.2002.384.doc.x

For peer review only - http://bmjopen.bmj.com/site/about/guidelines.xhtml

[33] Braun V , Clarke V . Using thematic analysis in psychology. Qual Res Psychol 2006;3:77–101.doi:10.1191/147808870

[34] Young S, Asherson P, Lloyd T, Absoud M, Arif M, Colley WA, Cortese S, Cubbin S, Doyle N, Morua SD, Ferreira-Lay P, Gudjonsson G, Ivens V, Jarvis C, Lewis A, Mason P, Newlove-Delgado T, Pitts M, Read H, van Rensburg K, Zoritch B and Skirrow C (2021) Failure of Healthcare Provision for Attention-Deficit/Hyperactivity Disorder in the United Kingdom: A Consensus Statement. Front. Psychiatry 12:649399. doi: 10.3389/fpsyt.2021.649399

Figure Caption

Figure 1: QbTest Consort chart

For peer review only

For peer review only - http://bmjopen.bmj.com/site/about/guidelines.xhtml

Figure 1: QbTest Consort chart

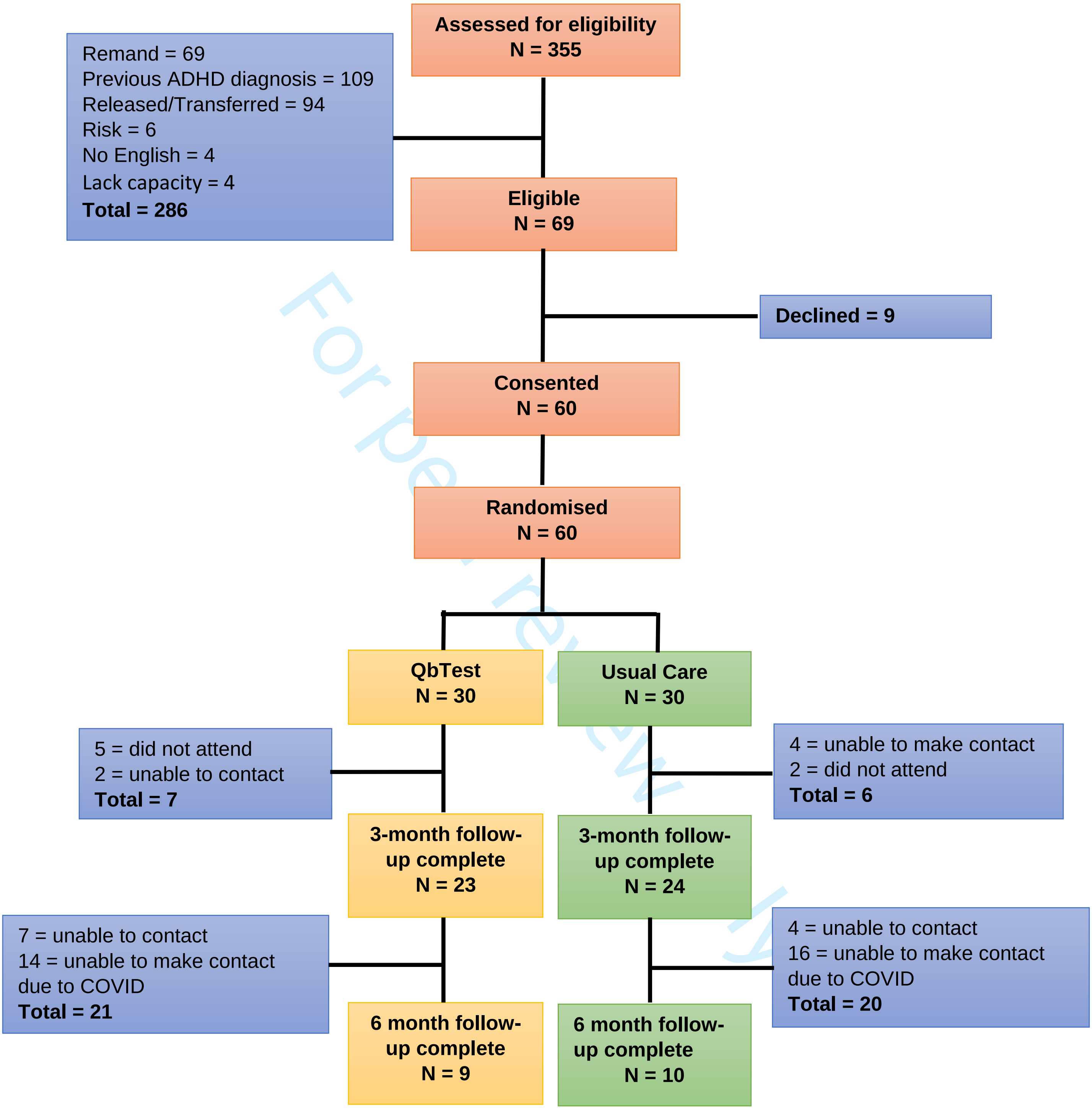

CONSORT 2010 checklist of information to include when reporting a randomised trial*

Section/Topic	Item No	Checklist item	Reported on page No
**Title and abstract**			
	1a	Identification as a randomised trial in the title	1
	1b	Structured summary of trial design, methods, results, and conclusions (for specific guidance see CONSORT for abstracts)	2-3
**Introduction**			
Background and objectives	2a	Scientific background and explanation of rationale	4
	2b	Specific objectives or hypotheses	5
**Methods**			
Trial design	3a	Description of trial design (such as parallel, factorial) including allocation ratio	5
	3b	Important changes to methods after trial commencement (such as eligibility criteria), with reasons	NA
Participants	4a	Eligibility criteria for participants	5
	4b	Settings and locations where the data were collected	5
Interventions	5	The interventions for each group with sufficient details to allow replication, including how and when they were actually administered	5-6
Outcomes	6a	Completely defined pre-specified primary and secondary outcome measures, including how and when they were assessed	6-8
	6b	Any changes to trial outcomes after the trial commenced, with reasons	NA
Sample size	7a	How sample size was determined	5
	7b	When applicable, explanation of any interim analyses and stopping guidelines	NA
Randomisation:			
Sequence generation	8a	Method used to generate the random allocation sequence	6
	8b	Type of randomisation; details of any restriction (such as blocking and block size)	6
Allocation concealment mechanism	9	Mechanism used to implement the random allocation sequence (such as sequentially numbered containers), describing any steps taken to conceal the sequence until interventions were assigned	6
Implementation	10	Who generated the random allocation sequence, who enrolled participants, and who assigned participants to interventions	6
Blinding	11a	If done, who was blinded after assignment to interventions (for example, participants, care providers, those	6

For peer review only

		assessing outcomes) and how	
	11b	If relevant, description of the similarity of interventions	NA
Statistical methods	12a	Statistical methods used to compare groups for primary and secondary outcomes	8
	12b	Methods for additional analyses, such as subgroup analyses and adjusted analyses	NA
**Results**			
Participant flow (a diagram is strongly recommended)	13a	For each group, the numbers of participants who were randomly assigned, received intended treatment, and were analysed for the primary outcome	8-9 (Figure 1)
	13b	For each group, losses and exclusions after randomisation, together with reasons	9-10 (Figure 1)
Recruitment	14a	Dates defining the periods of recruitment and follow-up	5
	14b	Why the trial ended or was stopped	5
Baseline data	15	A table showing baseline demographic and clinical characteristics for each group	Tables 1 and 2
Numbers analysed	16	For each group, number of participants (denominator) included in each analysis and whether the analysis was by original assigned groups	NA (feasibility study)
Outcomes and estimation	17a	For each primary and secondary outcome, results for each group, and the estimated effect size and its precision (such as 95% confidence interval)	NA (feasibility study)
	17b	For binary outcomes, presentation of both absolute and relative effect sizes is recommended	NA (feasibility study)
Ancillary analyses	18	Results of any other analyses performed, including subgroup analyses and adjusted analyses, distinguishing pre-specified from exploratory	NA (feasibility study)
Harms	19	All important harms or unintended effects in each group (for specific guidance see CONSORT for harms)	9
**Discussion**			
Limitations	20	Trial limitations, addressing sources of potential bias, imprecision, and, if relevant, multiplicity of analyses	3 and 12
Generalisability	21	Generalisability (external validity, applicability) of the trial findings	12-13
Interpretation	22	Interpretation consistent with results, balancing benefits and harms, and considering other relevant evidence	13
**Other information**			
Registration	23	Registration number and name of trial registry	3
Protocol	24	Where the full trial protocol can be accessed, if available	7

For peer review only - http://bmjopen.bmj.com/site/about/guidelines.xhtml

Funding	25	Sources of funding and other support (such as supply of drugs), role of funders	3

*We strongly recommend reading this statement in conjunction with the CONSORT 2010 Explanation and Elaboration for important clarifications on all the items. If relevant, we also recommend reading CONSORT extensions for cluster randomised trials, non-inferiority and equivalence trials, non-pharmacological treatments, herbal interventions, and pragmatic trials. Additional extensions are forthcoming: for those and for up to date references relevant to this checklist, see www.consort-statement.org.

For peer review only

BMJ Open

Using an objective computer task (QbTest) to aid the identification of Attention Deficit Hyperactivity Disorder (ADHD) in the Children and Young People Secure Estate (CYPSE): A feasibility randomised controlled trial

Journal:	*BMJ Open*
Manuscript ID	bmjopen-2022-064951.R1
Article Type:	Original research
Date Submitted by the Author:	01-Nov-2022

Complete List of Authors:	Chitsabesan, Prathiba; Pennine Care NHS Foundation Trust, Children and Young People's Research Unit<br>Hall, Charlotte; Institute of Mental Health, NIHR CLAHRC-East Midlands, Psychiatry<br>Carter, Lesley-Anne; The University of Manchester, Centre for Biostatistics, Division of Population Health, Health Services Research and Primary Care<br>Reeves, Mindy; The University of Manchester<br>Mohammed, Vaseem; The University of Manchester<br>Beresford, Bryony; University of York, Social Policy Research Unit<br>Young, Susan; University of Reykjavik,<br>Kraam, Abdullah ; Rotherham Doncaster and South Humber Mental Health NHS Foundation Trust, Children and Adolecent Mental Health<br>Trowse, Sally; Pennine Care NHS Foundation Trust<br>Wilkinson-Cunningham, Lloyd; Leeds Community Healthcare NHS Trust,<br>Lennox, Charlotte; University of Manchester, Manchester Academic Health Science Centre

**Primary Subject Heading**:	Mental health
Secondary Subject Heading:	Mental health
Keywords:	MENTAL HEALTH, Developmental neurology & neurodisability < PAEDIATRICS, Child & adolescent psychiatry < PSYCHIATRY

SCHOLARONE™
Manuscripts

I, the Submitting Author has the right to grant and does grant on behalf of all authors of the Work (as defined in the below author licence), an exclusive licence and/or a non-exclusive licence for contributions from authors who are: i) UK Crown employees; ii) where BMJ has agreed a CC-BY licence shall apply, and/or iii) in accordance with the terms applicable for US Federal Government officers or employees acting as part of their official duties; on a worldwide, perpetual, irrevocable, royalty-free basis to BMJ Publishing Group Ltd ("BMJ") its licensees and where the relevant Journal is co-owned by BMJ to the co-owners of the Journal, to publish the Work in this journal and any other BMJ products and to exploit all rights, as set out in our licence.

The Submitting Author accepts and understands that any supply made under these terms is made by BMJ to the Submitting Author unless you are acting as an employee on behalf of your employer or a postgraduate student of an affiliated institution which is paying any applicable article publishing charge ("APC") for Open Access articles. Where the Submitting Author wishes to make the Work available on an Open Access basis (and intends to pay the relevant APC), the terms of reuse of such Open Access shall be governed by a Creative Commons licence – details of these licences and which Creative Commons licence will apply to this Work are set out in our licence referred to above.

Other than as permitted in any relevant BMJ Author's Self Archiving Policies, I confirm this Work has not been accepted for publication elsewhere, is not being considered for publication elsewhere and does not duplicate material already published. I confirm all authors consent to publication of this Work and authorise the granting of this licence.

For peer review only

Using an objective computer task (QbTest) to aid the identification of Attention Deficit Hyperactivity Disorder (ADHD) in the Children and Young People Secure Estate (CYPSE): A feasibility randomised controlled trial

Authors and affiliations

1. Prathiba Chitsabesan[1,2],
2. Charlotte Lucy Hall[3],
3. Lesley-Anne Carter[4],
4. Mindy Reeves[5],
5. Vaseem Mohammed[5],
6. Bryony Beresford[6],
7. Susan Young[7],
8. Abdullah Kraam[8],
9. Sally Trwose[1],
10. Lloyd Wilkinson-Cunningham[9],
11. Charlotte Lennox[10]

Corresponding author- Prathiba Chitsabesan - pchitsabesan@nhs.net

1. Child and Adolescent Mental Health Service, Pennine Care NHS Foundation Trust, Ashton-under-Lyne, Lancashire, UK.

2. Division of Health Psychology and Social Care, Manchester Metropolitan University, Manchester, UK

3. NIHR MindTech MedTech Cooperative, Mental Health & Clinical Neurosciences, Institute of Mental Health, University of Nottingham, Nottingham, Nottinghamshire, UK

4. Centre for Biostatistics, Division of Population Health, Health Services Research and Primary Care, The University of Manchester, Manchester, UK

5. Medical School, The University of Manchester, Manchester, UK

6. Social Policy Research Unit, University of York, York, North Yorkshire, UK

7. Department of Clinical and Forensic Psychology, Psychology Services Limited, London, UK and Department of Psychology, Reykjavik University, Iceland

For peer review only - http://bmjopen.bmj.com/site/about/guidelines.xhtml

8. Child and Adolescent Mental Health Service, Rotherham Doncaster and South Humber Mental Health NHS Foundation Trust, Doncaster, UK

9. Research and Development, Leeds Community Healthcare NHS Trust, Leeds, UK

10. Faculty of Biology, Medicine and Health, School of Health, Division of Psychology and Mental Health, The University of Manchester, Manchester, UK

Abstract

Objectives: QbTest has been shown to improve time to decision/diagnosis for young people with Attention Deficit Hyperactivity Disorder (ADHD). The aim was to assess feasibility of QbTest for young people in prison.

Design: Single centre feasibility Randomised Controlled Trial (RCT), with 1:1 allocation. Concealed random allocation using an online pseudo-random list with random permuted blocks of varying sizes.

Setting: One Young Offenders Institution (YOI) in England.

Participants: 355 young people aged 15-18 years displaying possible symptoms of ADHD were assessed for eligibility, 69 were eligible to take part and 60 were randomised.

Intervention: QbTest - a computer task measuring attention, activity, and impulsivity.

Main outcome measures: Eligibility, recruitment and retention rates and acceptability of randomisation and trial participation.

Results: Of the 355 young people assessed for eligibility, 69 were eligible, and 60 were randomised (n = 30 QbTest plus usual care; n = 30 usual care alone). The study achieved the specified recruitment target. Trial participation and randomisation were deemed acceptable by the majority of participants. 78% of young people were followed up at 3-months, but only 32% at 6-months, although this was also affected by COVID restrictions. Secondary outcomes were mixed. Participants including clinical staff were mostly supportive of the study and QbTest, however some young people found QbTest hard and there were issues with implementation of the ADHD care pathway. There were no serious adverse events secondary to the study or intervention and no one was withdrawn from the study due to an adverse event.

Conclusions: With adaptations, a fully powered RCT may be achievable to evaluate the effectiveness of QbTest in the assessment of ADHD in the CYPSE, with time to decision (days) as the primary outcome measure. However, further programme developmental work is required to address some of the challenges highlighted prior to a larger trial.

For peer review only - http://bmjopen.bmj.com/site/about/guidelines.xhtml

Trial registration: ISRCTN17402196

Strengths and limitations

- The study has contributed to developing the evidence base in understanding barriers and facilitators to implementing QbTest within a secure estate setting.
- The study produced important feasibility information on eligibility, recruitment, attrition rates and collection of primary outcomes.
- The sample was obtained from only one site within the secure estate and includes boys only.
- While this was a pragmatic trial to assess feasibility, there were challenges identified in the usual care pathway and the number of diagnostic decisions within the follow up period was limited.
- COVID restrictions had an impact on delivery of usual care, access to the prison and collection of some of the outcome measures.

For peer review only

For peer review only - http://bmjopen.bmj.com/site/about/guidelines.xhtml

Background

Attention Deficit Hyperactivity Disorder (ADHD) is a common mental health disorder; signficanly greater in prevalence in the Children and Young People Secure Estate (CYPSE) up to 30.1% [1]; than the general population of young people 5% [2]. However, a recent report raised significant concerns about the number of young people in the CYPSE with undetected neurodisability, including ADHD, due to a lack of appropriate screening and assessment processes and training of staff [3].

A clinical assessment of ADHD requires integration of information including observation and reports from parents, teachers, and young people. This can be difficult to obtain for young people in the CYPSE. This approach is also reliant on subjective measures which can lead to a lack of reliability and consistency in the diagnosis of ADHD [4] or delays when there is a difference of opinion between informants [5]. All these factors can lead to delays in receiving a diagnosis and accessing evidence-based treatment. Early diagnosis and timely interventions reduce the risk of adverse long-term outcomes that are associated with ADHD such as antisocial behaviour, poor academic performance, and social functioning [6]. The social and economic burden of untreated ADHD on society is significant [7-9] and is estimated to be around £100,000 per case [10], although this does not include any criminal justice costs. There are effective treatments for ADHD including medication that can improve outcomes for young people [11,12]. One way of supporting the assessment for ADHD is the use of objective measures.

One objective measure is the continuous performance test (CPT). There are several variants of this but all measure vigilance and sustained attention. While CPT can demonstrate good sensitivity to ADHD and correlates well with symptoms [13], other studies have shown significant overlap in the performance of children with ADHD and typically developing children [14] and variability in intellectual ability may confound the interpretation of CPT performance in ADHD [15].

However, recent evidence suggests that combining a CPT with an objective measure of motor activity may add value in the clinical assessment of ADHD [16-17].

QbTest (Qbtech Ltd) combines a computerised CPT with an infrared camera to detect motor activity during the test and provides an objective standardised measurement of attention, impulsivity and activity, QbTest is highly correlated with blinded observer ratings of ADHD symptoms in placebo-controlled trials [18] and can help differentiate ADHD from other conditions [19].

For peer review only - http://bmjopen.bmj.com/site/about/guidelines.xhtml

In studies designed to assess ‘stand-alone’ diagnostic accuracy, QbTest has only moderate sensitivity and low specificity to ADHD [20]. Additionally, there are concerns about QbTest identifying ADHD in clinical samples [21]. However, these studies used QbTest independently of other clinical information. QbTest is not designed to act as a ‘stand-alone’ tool; the US FDA approved QbTest as a decision-aid tool to augment, but not replace, standard clinical assessment of ADHD by a trained practitioner [11].

A recently published single blind randomised control trial [22] demonstrated positive results for the role of Qbtest in supporting ADHD diagnosis alongside a routine ADHD assessment in a clinic referred sample. This assessment approach has been shown to be acceptable to both families and clinicians [23]. At 6 months, clinicians with access to the QbTest report were more likely to reach a diagnostic decision about ADHD, felt more confident in their decision and took less consultation minutes to reach the decision compared to those who had not seen a QbTest report. There was no difference in diagnostic accuracy. The authors concluded QbTest may increase the efficiency of ADHD assessment pathway allowing greater patient throughput with clinicians reaching diagnostic decisions faster without compromising diagnostic accuracy [22]. One suggested approach to using CPTs is in a step-wise approach to support the diagnostic process at a later stage when there is clinical uncertainty [24]. While this may be a feasible approach in community settings, it may delay diagnostic decision making and young people only remain in the secure estate for relatively short periods of time.

For peer review only

Aims and objectives

To assess the feasibility and acceptability of conducting a pragmatic RCT of QbTest in the assessment of ADHD in the CYPSE.

Methods

Trial Design

We conducted a single centre parallel two-group feasibility RCT with 1:1 individual participant allocation to QbTest plus usual care or usual care alone. Recruitment started 26 March 2019 and ended 27 February 2020. Six-month follow-up continued until October 2020. The primary aim of the study was to assess the feasibility and acceptability of conducting a pragmatic RCT of QbTest in the assessment of ADHD in the CYPSE. We were interested to evaluate whether

For peer review only - http://bmjopen.bmj.com/site/about/guidelines.xhtml

outcomes measures could be reliably collected at baseline and follow up and to help identify primary and secondary measures for a larger RCT.

Participants

The study took place in one Young Offenders Institution (YOI) accommodating boys aged 15-18 years in England. Participants were eligible if they answered a 'yes' to any of the ADHD symptoms on the CHAT assessment (Comprehensive Health Assessment Tool[1]). Participants were excluded if: they were on remand, did not speak English, had a previous or current diagnosis of ADHD, deemed a risk to either the researcher or staff, unable to provide informed consent (over 16 years) or parent/legal guardian consent was not received (under 16). Consent was obtained by the research assistant. The flow of participants is shown in figure 1. A sample size of 60 (30 to each group) were large enough to test the feasibility of the research procedures [25].

Insert figure 1 about here

Usual Care

Within the YOI, the expected usual care was as follows: young people identified with potential ADHD are offered an initial appointment by the neurodevelopmental lead, and initially assessed to decide whether the young person potentially has ADHD. This appointment was expected to be offered within a month of randomisation. If there were potential ADHD symptoms then an Assistant Mental Health Practitioner would commence the assessment by completing questionnaires, collecting a developmental history, and observing the young person. The time frame for this varies, but would aim to be completed within two months of randomisation. Once the history, observation and informant information are collected, another appointment would be made for the young person with the neurodevelopmental lead to review the information and either diagnose with ADHD or exclude diagnosis. If a diagnosis were made, then medication would be trialled, and the young person monitored for any improvements in presentation or side effects from medication.

[1] The CHAT is a health needs assessment used in the CYPSE at the point of admission. It assesses first night risk e.g. self-harm, followed by a comprehensive assessment of physical and mental health, substance use and neurodisability over the first 10 days of admission. The ADHD section on the CHAT contains symptom questions around concentration, restlessness/fidgeting, interrupting and difficult waiting turn.

For peer review only - http://bmjopen.bmj.com/site/about/guidelines.xhtml

Intervention – Usual care plus QbTest

Prior to the triage appointment by the neurodevelopmental lead, young people in the QbTest group were booked into the QbTest clinic. QbTest (Qbtech Ltd) is a computer task that measures three core aspects of ADHD: attention, impulsivity, and motor activity. Performance on the task provides information (via an immediate report) on the three symptom domains of ADHD and a 'summary score' based on deviation from a normative data set (for age and gender). The healthcare team conducted the QbTest and used the information from the QbTest report in conjunction with the clinical information to inform their decision whether the young person had ADHD or not.

Randomisation

Randomisation was completed by the research assistant and achieved by means of concealed random allocation using an online pseudo-random list hosted by Sealedenvelope.com with random permuted blocks of varying sizes.

Blinding

Given the nature of QbTest, it was not possible to blind participants or those involved in delivering the intervention. Participants and research team were blind to allocation at the time of the baseline measures being completed which occurred before randomisation. It was not possible to maintain the field researcher being blind to trial arm allocation because the researcher and staff delivering the intervention were both working within the confines of the YOI. Only the statistician (LAC) who was assessing the outcomes was blind to trial arm allocation.

Primary Outcomes

The following outcome measures and data were collected:

- Eligibility rate recorded as the number of eligible young people against the total number of young people assessed for eligibility (i.e., identified with ADHD needs on the CHAT);
- Recruitment rate recorded as the number of young people who consent to participate against the number of eligible young people;
- Acceptability of randomisation recorded as the number of young people randomised;
- Acceptability of trial participation recorded as the number of eligible young people who dropped out after receiving allocation;

For peer review only - http://bmjopen.bmj.com/site/about/guidelines.xhtml

- Retention rate recorded as the number of young people who were randomised that remain in the study until the end of follow up at 6 months (target of 50% at 6 months in both trial arms).

Secondary Outcomes

The secondary outcome measures selected included measures of ADHD symptoms, quality of life and service use. Other key data collected such as the number of appointments and days until diagnosis and number of behavioural episodes were also key outcome data that would be relevant to a larger study in measuring the impact of the intervention arm (QbTest) and identifying differences between the two groups (usual care vs usual care and QbTest group). The following outcome measures and data were collected at baseline, 3 and 6 months, unless otherwise stated:

Completed by the young person with support from the research assistant

- Behaviour measured using the Strengths and Difficulties Questionnaire (SDQ) [26]. The SDQ is a well validated and commonly used brief screening questionnaire of children's emotions and behaviours through 5 scales (e.g., emotional; conduct problems; hyperactivity/inattention; peer relationship problems and prosocial) scored 0-10. Scores are categorised from 'close to average' (80% of population) to 'very high' (5% of population).
- ADHD symptoms were measured using the Brief Barkley ADHD Rating Scale (B-BAARS) [27]. The Brief-Barkley Adult ADHD Rating Scale (B-BAARS) has been developed as a brief assessment to screen for ADHD in adults in the criminal justice system and has been shown to accurately predict the likelihood of receiving a clinical diagnosis using DSM-V criteria.
- Health-related quality of life was measured using the Child Health Utility Instrument (CHU-9D) [28] [2]. The CHU-9D is a quality of life measure containing 9 main dimensions (worried, sad, pain, tired, annoyed, schoolwork/homework, sleep, daily routine, and activities), each with 5 increasing levels of severity/impairment.
- Contact with services was measured using the modified Client Service Receipt Inventory (CSRI) [29]. It's allows resource use patterns to be described and support costs to be estimated using an appropriate unit cost.
- .At 3 months feedback from young people receiving the QbTest using the QbTest opinion questionnaire.

[2] Only reported as level of missing data.

For peer review only - http://bmjopen.bmj.com/site/about/guidelines.xhtml

Completed by the clinical team

- The Children's Global Assessment Scale (C-GAS) [30] is a rating of functioning for young people 6-17years of age. The young person is given a single score between 1 and 100 based on a clinician's assessment of the young person's psychological and social functioning. The score puts them in one of ten categories that range from 'extremely impaired' (1-10) to 'doing very well' (91-100).
- Number of appointments until confirmed ADHD diagnosis/exclusion of ADHD and number of days until a decision was reached was recorded on a pro-forma completed by clinicians after each appointment with the young person.

Completed by the teacher

- The Swanson, Nolan, and Pelham Rating Scale (SNAP-IV) is a widely used scale that measures the core symptoms of attention deficit hyperactivity disorder (ADHD) [31]. Teachers are asked to rate 26 items on a 4point scale.

Completed by the research assistant from health and prison records

- Number of recorded behavioural incidents were collected using prison information.
- Numbers of young people agreeing to sit the QbTest and the number who completed the QbTest was recorded.

Qualitative interviews with young people and staff

- At 3 months participants were interviewed using a semi-structured interview schedule about the acceptability of randomisation, acceptability of outcome measures and for those in the QbTest group acceptability of the QbTest. Purposive sampling was used to select participants for interview including consideration of age, completion of the QbTest and scores on the QbTest opinion questionnaire.
- At the end of the study health professionals and CYPSE staff were interviewed on the acceptability and feasibility of administering and implementing QbTest within usual assessment practices as well as facilitators and barriers to using the QbTest and reasons for non-completion.

See study protocol [32] for details of how data collection was conducted.

Data Analysis

For peer review only - http://bmjopen.bmj.com/site/about/guidelines.xhtml

For peer review only

As a feasibility study, data analysis was mainly descriptive [33]. All measures were summarised by group across follow-up time with mean and standard deviation for normally distributed data, median for skewed variables and frequency (percentage) for categorical data. All statistical analysis were conducted using Stata v.15.

Interviews were conducted and recorded by the researcher using an encrypted Dictaphone. All qualitative data were analysed using thematic analysis [34] with the aid of NVivo. Data were coded inductively into themes, creating a detailed coding scheme, allowing for the investigation of emergent patterns between individual codes and boarder emergent themes.

Patient and Public Involvement (PPI)

PPI was embedded in many aspects of the study including; the development of the funding application and shaping the study objectives and outcome measures. The burden of the intervention for young people had been previously assessed through a local community audit. Within the project we supported the development of a PPI group within the YOI and they met on a quarterly basis, facilitated by the research assistant. This group helped develop our Patient Information Sheet and advised on recruitment and data collection including the need for the researcher to be flexible in their approach eg seeing young people on the unit rather than the young people attending healthcare. COVID significantly impacted our ability to continue this group and as a result, our PPI group were not involved in the analysis or dissemination stages of the project as we had planned. However, our PPI representatives (two parents of young people with ADHD) have helped us interpret the qualitative data and write lay summaries and infographics for staff and young people. This will be disseminated to staff through a webinar and written summary and to the young people through the healthcare team.

Results

A total of 60 young people were randomised 30 to QbTest plus usual care (intervention) and 30 to usual care (control). Table 1 shows the demographics of the 60 boys randomised to the study. Of the 60 young people a sub-sample of 11 young people (6 QbTest v 5 usual care) were interviewed. The research assistant and five members of staff were also interviewed, including all those who were delivering QbTest and staff involved in supporting the implementation.

Table 1: Baseline demographic information

For peer review only - http://bmjopen.bmj.com/site/about/guidelines.xhtml

		QbTest (n = 30)		Usual Care (n = 30)	
		n	%	n	%
**Age**					
	**16**	6	20.0	3	10
	**17**	8	26.7	11	36.7
	**18**	15	50.0	16	53.3
	**Missing**	1	3.3	0	0
**Ethnicity**					
	**White**	23	76.7	24	80.0
	**Other**	6	20.0	6	20.0
	**Missing**	1	3.3	0	0
**Accommodation**					
	**House or flat**	20	66.7	25	83.3
	**Other**	9	30.0	5	16.7
	**Missing**	1	3.3	0	0
**Lived with**					
	**Parent(s)**	15	50.0	21	70.0
	**Other**	13	43.3	9	30.0
	**Missing**	2	6.7	0	0
**Education**					
	**Mainstream**	6	20.0	6	20.0
	**Pupil Referral Unit**	3	10.0	5	16.7
	**None**	20	66.7	17	56.7
	**Other**	0	0	2	6.7
	**Missing**	1	3.3	0	0

Primary Outcomes

Eligibility rate

Participant flow (see Figure 1) showed that over 12 months we assessed 355 young people for eligibility. The numbers and reasons for ineligibility were: 69 (19%) on remand, 109 (30%) had a previous diagnosis of ADHD, 94 (26%) were released or transferred before they could be approached, 6 (2%) were deemed too high risk, 4 (1%) were not fluent in English as a language and 4 (1%) were deemed to lack capacity. This gave us an eligibility rate of 19.4% (69/355).

Recruitment rate

Of the 69 young people who were eligible, 60 (87%) consented to participate in the study. For the nine participants who did not consent, their reason was either they did not feel they had ADHD or that they were leaving prison soon. Our initial estimates were to achieve an average recruitment rate of five randomisations a month (60 young people over 12 months) which was successfully achieved.

Acceptability of randomisation and trial participation

For peer review only - http://bmjopen.bmj.com/site/about/guidelines.xhtml

Of the 60 young people who consented to take part all agreed to be randomised and no young people refused to continue in the study after randomisation (see Figure 1).

Retention rate

Of the 60 young people 47 were followed up at 3-months, a retention rate of 78% which exceeded our target. Our aim was to successfully follow up at least 50% at 6 months. However, we were only able to follow up 19 of the 60 young people; a retention rate of 32%. Half of the 6-month follow-ups were due after March 2020 and therefore were significantly impacted by COVID as we were not able to access the YOI from March 2020 for 8months.

There were no serious adverse events secondary to the study or intervention and no one was withdrawn from the study due to an adverse event.

Secondary Outcomes

Of the 30 young people randomised to receive QbTest all agreed to sit the QbTest. At the end of the recruitment window four young people could not undertake their QbTest due to the COVID lockdown in the prison. For the remaining 26 young people, a total of 6 (23%) did not complete their QbTest: for three young people (12%) the appointment was not attended due to prison resources (staffing to accompany the young person), two (8%) young people were released/transferred before the QbTest could take place and one young person (4%) was unable to sit the QbTest due to concerns from the clinical team regarding increased risk of self-harm at the time. The median number of days between randomisation and QbTest was 42 (IQR = 26 – 93; min = 1; max = 195).

Table 2 shows the descriptive statistics for the questionnaires collected at baseline, 3 and 6 months for the two trial arms from the young people.

For peer review only

For peer review only - http://bmjopen.bmj.com/site/about/guidelines.xhtml

Table 2: Descriptive statistics for secondary outcome measures

		Baseline (n=60)				3 months (n=47)				6 months (n=19)			
		QbTest		Usual Care		QbTest		Usual Care		QbTest		Usual Care	
		N	%	n	%	n	%	n	%	n	%	n	%
**SDQ Total cut-off**													
	Close to Average	7	23.3	5	16.7	2	6.7	4	13.3	2	6.7	3	10.0
	Slightly Raised	4	13.3	8	26.7	0	0	5	16.7	0	0	4	13.3
	High	2	6.7	5	16.7	4	13.3	1	3.3	0	0	1	3.3
	Very High	16	53.3	12	40.0	7	23.3	7	23.3	7	23.3	1	3.3
	Missing	1	3.3	0	0	17	56.7	13	43.3	21	70.0	21	70.0
**B-BAARS**													
Likelihood of receiving	Yes	21	70.0	23	76.7	10	33.3	12	40.0	7	23.3	4	13.3
a clinical Diagnosis	No	8	26.7	7	23.3	3	10.0	6	20.0	2	6.7	4	13.3
(DSM V criteria)	Missing	1	3.3	0	0	17	56.7	12	40.0	21	70.0	22	73.3
**SNAP[a]**													
	Missing	26	86.7	27	90	30	100	30	100	30	100	30	100
**CHU-9D[a]**													
	Missing	2	6.7	0	0	17	56.7	13	43.3	21	70.0	22	73.3
**C-GAS[b]**													
	Some noticeable problems	3	10.0	0	0	0	0	0	0	0	0	0	0
	Some problems	3	10.0	0	0	1	3.3	0	0	0	0	0	0
	Doing all right	1	3.3	1	3.3	0	0	2	6.7	0	0	1	3.3
	Doing well	0	7	2	6.7	0	0	2	6.7	0	0	0	0
	Missing	23	76.7	27	90.0	29	96.7	26	86.7	30	100	29	96.7
**Service Use (CSRI)**													
Emergency	Yes	4	13.3	8	26.7	2	6.7	1	3.3	2	6.7	1	3.3
Attendance	No	23	76.7	22	73.3	11	36.7	16	53.3	7	23.3	7	23.3
Physical Health	Missing	3	10.0	0	0	17	56.7	13	43.3	21	70.0	22.0	73.3
Planned	*Yes*	1	3.3	0	0	0	0	0	0	0	0	0	0
Admission	*No*	26	86.7	30	100	13	43.3	17	56.7	9	30.0	8	26.7
Physical Health	*Missing*	3	10.0	0	0	17	56.7	13	43.3	21	70.0	22	73.3

For peer review only - http://bmjopen.bmj.com/site/about/guidelines.xhtml

Outpatient Appointments Physical Health	Yes	2	6.7	2	6.7	1	3.3	4	13.3	0	0	1	3.3
	No	25	83.3	28	93.3	12	40.0	13	43.3	9	30.0	7	23.3
	Missing	3	10.0	0	0	17	56.7	13	43.3	21	70.0	22	73.3
Missed Appointments	Yes	6	20.0	5	16.7	3	10.0	1	3.3	1	3.3	0	0
	No	21	70.0	25	83.3	10	33.3	16	53.3	8	26.7	8	26.7
	Missing	3	10.0	0	0	17	56.7	13	43.3	21	70	22	73.3
Emergency Attendance Menta Health	Yes	1	3.3	2	6.7	0	0	0	0	0	0	0	0
	No	26	86.7	28	93.3	13	43.3	17	56.7	9	30.0	8	26.7
	Missing	3	10.0	0	0	17	56.7	13	43.3	21	70.0	22	73.3
Planned Admission Mental Health	Yes	0	0	0	0	0	0	0	0	0	0	0	0
	No	24	80.0	30	100.0	13	43.3	16	53.3	9	30.0	8	26.7
	Missing	6	20.0	0	0	17	56.7	14	46.7	21	70.0	22	73.3
Missed Appointments Mental Health	Yes	1	3.3	1	3.3	1	3.3	2	6.7	0	0	0	0
	No	25	83.3	29	96.7	12	40.0	15	50	9	30.0	7	23.3
	Missing	4	13.3	0	0	17	56.7	13	43.3	21	70.0	23	76.7
**Number of Service Contacts (CSRI)**													
Physical Health Contacts	Median	3		2		4		1		2		1	
	IQR	1-8		1-4		1-8		0-6		2-3		0-12	
	Min Max	0, 47		0, 73		0, 88		0, 14		0, 6		0, 51	
	n	27		30		13		17		9		8	
Mental Health Contacts	Median	6		4		2		5		2		0.5	
	IQR	2-20		1-6		2-4		4-12		1-5		0-1.5	
	Min Max	0, 89		0,333		0, 18		0, 24		0,37		0,5	
	n	27				13		17		9		8	
Criminal Justice Contacts	Median	9		8.5		4		4		2		5	
	IQR	3-23		5-20		3-6		3-6		1-4		2.5-26	
	Min Max	1,107		1,106		0,65		0,16		0,137		2,60	
	n	27		30		13		17				8	
**Number of Incidents**													
	Median	1		1		2		2		3.5		7	
	IQR	0-2		0-2		1-5		0-6.5		1-4		2-7	
	Min Max	0, 12		0, 19		0, 12		0, 19		1, 7		0, 8	
	n	29		29		9		12		6		5	

a -missing data presented only

b- CGAS -has 10 categories from extremely impaired to doing very well- relevant categories presented only

For peer review only - http://bmjopen.bmj.com/site/about/guidelines.xhtml

The amount of missing data for all outcomes are shown in Table 2. Of those contactable for the 3 month follow up, 64%-66% completed the SDQ, B-BAARRS and CHU-9D. Completion rates of the 6 month measures were higher, however only 19 participants were contactable. The C-GAS and SNAP were poorly completed across all time points. As the study aimed to assess the feasibility of collecting outcome measures, we have not made comparisons of the data between intervention arms and over time points.

Of the 60 young people, a total of 32 (53%) received at one appointment and three (5%) had two appointments. Therefore, a total of 25 young people (42%) did not receive any appointment within the 6-month follow-up window. Some young people did have appointments, which fell outside of the 6-month window. Appointments had been offered to some young people but were not attended (n = 53).

The low number of appointments offered and attended within the 6month follow up period impacted on the number of decisions being made. We recorded evidence of 14 decisions across the 60 participants, 8 in the QbTest group and 6 in the usual care group (27% event rate vs 20%). All these decisions were exclusions of ADHD; there were no diagnoses of ADHD for any young people.

Data from the qualitative interviews suggested that most of the young people had some understanding of the randomisation process and were able to explain what had happened. Most of the young people said that they were happy or were not upset about being allocated to either group. There were however two young people who expressed stronger feelings about the group to which they were allocated. One was angry about being allocated to the QbTest group because they did not like the test as it took too long, and the other was allocated to usual care but wanted to receive QbTest as they thought they had ADHD. The young people were all happy with being involved in the research and one young person said that they would be happy to recommend the study to others.

Of the 20 young people who had received QbTest, 10 completed the QbTest questionnaire. Table 3 shows an overview of the data.

Table 3: Descriptive statistics for the QbTest feedback questionnaire

Item	Disagree	Neither	Agree
The QbTest results helped me understand my symptoms	2	6	2
The QbTest results were difficult to understand	1	7	2
I fully understood the purpose of the QbTest	1	0	9
I found the assessment very stressful	1	0	9

For peer review only - http://bmjopen.bmj.com/site/about/guidelines.xhtml

The task took too long to complete	0	1	9
I found the task difficult to complete	1	1	8
Overall the experience was useful	3	5	2
I found the chair very uncomfortable	3	5	2
The QbTest helped me understand the changes in my symptoms since my previous appointment[a]	1	7	1
I found the QbTest much easier to complete when on meds[b]	**0**	**6**	**0**
When the clinician talked through the output with me, it helped me understand how s/he had reached their diagnosis[a]	**0**	**6**	**3**
When the clinician talked me through the output, it helped me understand how they had reached their decision about medication	0	6	2

a = missing data for one young person b = missing data for four young people;

The questionnaire feedback suggests that young people may not have seen a significant benefit from undertaking the QbTest although not all had received feedback on the test at the time they completed the QbTest questionnaire. Data from the qualitative interviews also suggested that while some young people had been fine at the beginning of the test, they had found the process long and at times boring or felt exhausted by the end. One young person had to repeat the test and admitted feeling cross by this although another said he would recommend QbTest to other young people. At the time of the interview, not all young people had received the outcome of the test and ADHD assessment. One young person reported feeling excited by the possibility of a diagnosis and happy that he would be receiving help. Another young person was told that he did not have ADHD after the QbTest assessment but was unhappy with this as he did not feel this was correct.

Feedback from staff was generally more positive although they identified some challenges in using QbTest within a YOI setting. Initially there had been some concerns because of the equipment (laptop and camera) and IT system needed but it was felt that it was easy to address. The main difficulties appeared to be finding an appropriate room for the equipment which was quiet for the testing environment. While it had been planned that all QbTests would be undertaken in the same room and the equipment set up; staff did have to move to other rooms on occasion. One member of staff said that it was easy to carry the equipment around and set up in another location if needed. There were problems with the prison staff facilitating the appointments in healthcare (e.g., challenges in bringing the young people over to healthcare for their appointment). The healthcare staff often had to offer multiple appointments for the young person to attend to take the QbTest.

The staff members interviewed were of the opinion that QbTest was helpful in assessing ADHD and one staff member also reported that it helped both the young person and staff understand the young person's behaviours better. Staff felt that the QbTest could be helpful

For peer review only - http://bmjopen.bmj.com/site/about/guidelines.xhtml

in improving waiting times and worked well alongside the ADHD clinical assessment. One staff member felt the QbTest was more objective and there was less bias than self-report measures. All the staff interviewed felt that the QbTest should be used in other sites as there were many advantages of using the QbTest. One staff member added that the QbTest was helpful in the assessment of complex young people, where there might be concerns about co-morbid diagnosis.

Discussion, Limitations and Lessons Learnt

Findings

Despite the impact of COVID restrictions, this study has provided important information regarding the feasibility of introducing QbTest into the secure estate and conducting a larger trial. Our primary outcomes were largely achieved; estimates of eligibility were broadly accurate, consent rates were high, we reached our recruitment target on time, randomisation and trial participation was high. Not all participants randomised to QbTest, undertook the test; primarily due to the impact of COVID restrictions or staffing resources. We were able to follow-up young people, but this was easier over 3 months than 6 months and where the young people stayed within the YOI. Our secondary outcomes were more mixed; completion of outcome measures by young people was good overall at 3months and 6months where the young person remained in the secure estate or was contactable, but much poorer across all timepoints for measures completed by clinical staff and teachers. Only 23% of young people received a decision on diagnosis while they were within the secure estate which were all exclusion of ADHD. This is significantly lower than the number of diagnostic decisions made at 6months from a community based study (76% with QbTest plus usual ADHD care pathway and 50% with the usual ADHD care pathway alone) [22]. This may reflect both the complexity of the needs of young people in the secure estate and the challenges in undertaking assessments within this environment.

Participants (both young people and staff) were largely supportive of the study and the role of QbTest. We know from clinical practice, that young people can find QbTest challenging as it tests areas that they struggle with. This was evident from the questionnaire feedback and the interviews. However, this has also been reported in other studies and is experienced in clinical practice [23] and did not prevent young completing the test once they had started.

Strengths and Weaknesses

For peer review only - http://bmjopen.bmj.com/site/about/guidelines.xhtml

The study has contributed to developing the evidence base in understanding barriers and facilitators to implementing QbTest within a secure estate setting and has produced important feasibility information on eligibility, recruitment, attrition rates and collection of primary and secondary outcomes.

However, there were a number of limitations. The sample was obtained from only one site within the secure estate and includes boys only. Similar to the AQUA trial [22] we allowed the clinical team to complete QbTest at a time that suited them. Although we had suggested that it would be ideal for the clinical team to undertake the QbTest within two weeks from randomisation the average number of days between randomisation and QbTest was much longer. Also, from the appointment data it suggested that not all young people were receiving usual care within the timeframes described by the clinical team at the start of the study and many young people did not receive any appointments. Young people not receiving appointments were across both trial arms and throughout recruitment, although it was exacerbated by the impact of COVID-19. Staffing shortages, appointments not being attended, short sentences and the rigidity of the prison regime were also contributing factors to fewer diagnostic decisions being made. Recently a consensus group of UK experts highlighted the issues within current ADHD care pathways [35], given the complexity of the CYPSE it is likely that these issues are exacerbated.

Conclusion

Aspects of this feasibility trial demonstrated that a larger RCT trial of the effectiveness of QbTest within the CYPSE could be feasible, with time to decision (days) as the primary outcome measure and secondary measures completed by the young person. However, features of the intervention implementation were mixed, partly exacerbated by the impact of COVID. The study highlighted challenges with the delivery of the usual ADHD care pathway within the secure estate which has implications for clinicians and commissioners. Prior to progressing to a trial, additional programme developmental work would need to be undertaken with staff working in the secure estate. This would involve the review of the usual care ADHD pathway to understand barriers to timely delivery and the development of a standardised ADHD care pathway across the CYPSE as well as strategies to improve collection of some secondary outcome measures. This would aim to increase the number of appointments young people have with healthcare teams and subsequently decisions on ADHD status, enabling a better estimate of a time to decision for usual care time and the required sample size for a large multi-site RCT.

For peer review only - http://bmjopen.bmj.com/site/about/guidelines.xhtml

Contributors

PC: Chief Investigator, conceptualisation, supervision, methodology, writing and editing; CLH: Conceptualisation, methodology, writing – review and editing; LAC: Statistician, conceptualisation, methodology, analysis, writing – review and editing; BB: Conceptualisation, methodology, writing – review and editing; SY: Conceptualisation, methodology, writing – review and editing; AK: Conceptualisation, methodology, writing – review and editing; ST: PPI representative, analysis, writing – review and editing; LWC: Researcher, data collection, writing – review and editing; MR: Researcher, data collection, data entry, analysis, writing – review and editing; VM: Researcher, data collection, data entry, analysis, writing – review and editing; CL: Project Manager, conceptualisation, methodology, data entry, analysis, writing and editing;

Acknowledgements

The active involvement of all young people and staff who participated within this research study and provided advice is gratefully acknowledged.

Disclaimer

The views expressed are those of the author(s) and not necessarily those of the NHS, the NIHR of the Department of Health. The study sponsor and funders have no role in study design, including collection, management, analysis and interpretation of data; writing of the report and the decision to submit the report for publication

Competing interests

The authors declare that they have no competing interests.

Funding

The study is funded by the NIHR RfPB (Grant number: PB-PG-1216-20007).

Availability of data and material

No additional data is available due to the population of research participants involved in this study.

For peer review only - http://bmjopen.bmj.com/site/about/guidelines.xhtml

Ethics Approval

All participants (or their parent or legal guardian) gave written informed consent to participate in the study. Ethical approval for the study was obtained from the Health and Research Care Wales; REC reference 18/WA/0347 and IRAS project ID 238947.

References

[1] Young S, Moss D, Sedgwick O, et al. A meta-analysis of the prevalence of attention deficit hyperactivity disorder in incarcerated populations. *Psychological Medicin*e 2015, 45(2):247-58. doi: 10.1017/S0033291714000762.

[2] Fazel S, Doll H, Langstrom N. Mental disorders among adolescents in juvenile detention and correctional facilities: a systematic review and metaregression analysis of 25 surveys. *Journal of the American Academy of Child and Adolescent Psychiatry* 2008, 47: 1010–1019.

[3] Hughes N, Williams H, Chitsabeasan P. Nobody made the connection: The prevalence of neurodisability in young people who offend. The Children's Commissioner 2012 https://www.childrenscommissioner.gov.uk/sites/default/files/publications/Nobody%20made%20the%20connection.pdf

[4] Ogundele MO, Ayyash HF, Banerjee S. Role of computerised continuous performance task tests in ADHD. *Progress in Neurology and Psychiatry* 2011, 15: 8-13. https://doi.org/10.1002/pnp.198

[5] Kovshoff H, Williams S, Vrijens M, et al. The decisions regarding ADHD management (DRAMa) study: uncertainties and complexities in assessment, diagnosis and treatment, from the clinician's point of view. *European Child & Adolescent Psychiatry* 2012, 21(2):87–99.

[6] Shaw M, Hodgkins P, Caci H, et al. A systematic review and analysis of long-term outcomes in attention deficit hyperactivity disorder: effects of treatment and non-treatment. *BMC Medicine* 2012, 10, 99.

[7] Biederman J, Petty C, Clarke A, et al. Predictors of persistent ADHD: An 11-year follow-up study. *Journal of Psychiatry Research* 2010, 45: 150-155.

[8] Arnold LE, Hodgkins P, Kahle J, et al. Long-term outcomes of ADHD: Academic achievement and performance. Journal of Attention Disorders 2020, 24(1) 73–85 DOI: 10.1177/1087054714566076 journals.sagepub.com/home/jad

For peer review only - http://bmjopen.bmj.com/site/about/guidelines.xhtml

[9] D'Amico F, Knapp M, Beecham J, et al. Use of services and associated costs for young adults with childhood hyperactivity/conduct problems: 20-year follow up. *The British Journal of Psychiatry* 2014, 204: 441-447.

[10] Khong,B. The lifetime costs of attention deficit hyperactivity disorder (ADHD). Centre for Mental Health 2014 https://www.centreformentalhealth.org.uk/costs-of-adhd

[11] National Institute for Health and Care Excellence (2018). Attention deficit hyperactivity disorder: diagnosis and management (NG87). National Institute for Health and Care Excellence. https://www.nice.org.uk/guidance/NG87

[12] National Institute for Health and Care Excellence (2013). Attention deficit hyperactivity disorder (QS39). National Institute for Health and Care Excellence. https://www.nice.org.uk/guidance/qs39

[13] Epstein JN, Erkanli A, Conners CK, et al. Relations between continuous performance test performance measures and ADHD behaviors. *Journal of Abnormal Child Psychology*, 2013, 31: 543–554.

[14] Zelnik N, Bennett-Back O, Miari W, et al. Is the test of variables of attention reliable for the diagnosis of attention-deficit hyperactivity disorder (ADHD)? *Journal of Child Neurology*, 2012, 27: 703–707.

[15] Park MH, Kweon YS, Lee SJ, et al. Differences in performance of ADHD children on a visual and auditory continuous performance test according to IQ. *Psychiatry Investigation* 2011, 8: 227–233.

[16] Hall CL, Valentine A Z, Groom MJ, et al. The clinical utility of the continuous performance test and objective measures of activity for diagnosing and monitoring ADHD in children: A systematic review. *European Child and Adolescent Psychiatry* 2016, 25 (7): 677-699.

[17] Gilbert H, Qin L, Li D, Zhang X, et al. Aiding the diagnosis of AD/HD in childhood: Using actigraphy and a continuous performance test to objectively quantify symptoms. *Research in Developmental Disabilities* 2016, 59: 35–42.

[18] Wehmeier PM, Schacht A, Wolff C, et al. Based Continuous Performance Test Combined with an Infra-Red Motion-tracking Device. *Journal of Child and Adolescent Psychopharmacology* 2011, 21 (5): 433-444.

[19] Vogt C, Shameli A. Assessments for attention-deficit hyperactivity disorder: use of objective measurements. *The Psychiatrist* 2011, 35: 380-383.

For peer review only - http://bmjopen.bmj.com/site/about/guidelines.xhtml

[20] Hult N, Kadesjö J, Kadesjö B, et al. ADHD and the QbTest: Diagnostic Validity of QbTest. *Journal of attention disorders* 2018, *22*(11): 1074–1080. https://doi.org/10.1177/1087054715595697

[21] Adamou M, Jones SL, Marks L, et al. Efficacy of Continuous Performance Testing in Adult ADHD in a Clinical Sample Using QbTest. *Journal of attention disorders* 2022, 10870547221079798. Advance online publication. https://doi.org/10.1177/10870547221079798

[22] Hollis C, Hall CL, Guo B, et al. The AQUA Trial Group. The impact of a computerised test of attention and activity (QbTest) on diagnostic decision-making in children and young people with suspected attention deficit hyperactivity disorder: single-blind randomised controlled trial. *Journal of Child Psychology and Psychiatry* 2018, 59(12):1298-1308.

[23] Hall CL, Valentine AZ, Walker GM, et al. Study of user experience of an objective test (QbTest) to aid ADHD assessment and medication management: a multi-methods approach. *BMC Psychiatry* 2017,17: 66. https://doi.org/10.1186/s12888-017-1222-5

[24] Jarrett MA, Meter AV, Youngstrom EA, et al. Evidence-Based Assessment of ADHD in Youth Using a Receiver Operating Characteristic Approach. *Journal of clinical child and adolescent psychology : the official journal for the Society of Clinical Child and Adolescent Psychology, American Psychological Association, Division* 2018, 53, *47*(5): 808–820. https://doi.org/10.1080/15374416.2016.1225502

[25] Billingham SA, Whitehead AL, Julious SA. An audit of sample sizes for pilot and feasibility trials being undertaken in the United Kingdom registered in the United Kingdom Clinical Research Network database. *BMC Medical Research Methodology* 2013, 13, 104.

[26] Goodman R. The Strengths and Difficulties Questionnaire: A research note. *Journal of Child Psychology and Psychiatry* 1997, 38: 581–586.

[27] Young S, González RA, Mutch L, et al. Diagnostic accuracy of a brief screening tool for attention deficit hyperactivity disorder in UK prison inmates. *Psychological Medicine* 2011, 46: 1449-1458.

[28] Stevens K. Assessing the performance of a new generic measure of health-related quality of life for children and refining it for use in health state valuation. *Applied Health Economics Health Policy* 2011, 9: 57–69.

[29] Beecham J, Knapp M. Costing psychiatric interventions, in G. Thornicroft (ed.) Measuring Mental Health Needs, Gaskell, 2nd edition, 2011, 200-224.

For peer review only - http://bmjopen.bmj.com/site/about/guidelines.xhtml

[30] Shaffer N, Gould MS, Brasic J. A Children's Global Assessment Scale (CGAS). *Archives of General Psychiatry* 1983, 40: 1228-1231.

[31] Swanson JM, Kraemer HC, Hinshaw SP, et al. Clinical relevance of the primary findings of the MTA: success rates based on severity of ADHD and ODD symptoms at the end of treatment. *Journal of the American Academy of Child and Adolescent Psychiatry* 2001, 40, 168-79.

[32] Lennox C, Hall CL, Carter L, et al. FACT: a randomised controlled trial to assess the feasibility of QbTest in the assessment process of attention deficit hyperactivity disorder (ADHD) for young people in prison—a feasibility trial protocol *BMJ Open* 2020;10:e035519. doi: 10.1136/bmjopen-2019-035519

[33] Lancaster GA, Dodd S, Williamson PR. Design and analysis of pilot studies: recommendations for good practice. *J Eval Clin Prac* 2004, 10: 307–12. doi:10.1111/j.2002.384.doc.x

[34] Braun V, Clarke V. Using thematic analysis in psychology. *Qual Res Psychol* 2006, 3:77–101.doi:10.1191/147808870

[35] Young S, Asherson P, Lloyd T, et al. Failure of Healthcare Provision for Attention-Deficit/Hyperactivity Disorder in the United Kingdom: A Consensus Statement. Front. Psychiatry 2021, 12:649399. doi: 10.3389/fpsyt.2021.649399

Figure Caption

Figure 1: QbTest Consort chart

For peer review only

For peer review only - http://bmjopen.bmj.com/site/about/guidelines.xhtml

Figure 1: QbTest Consort chart

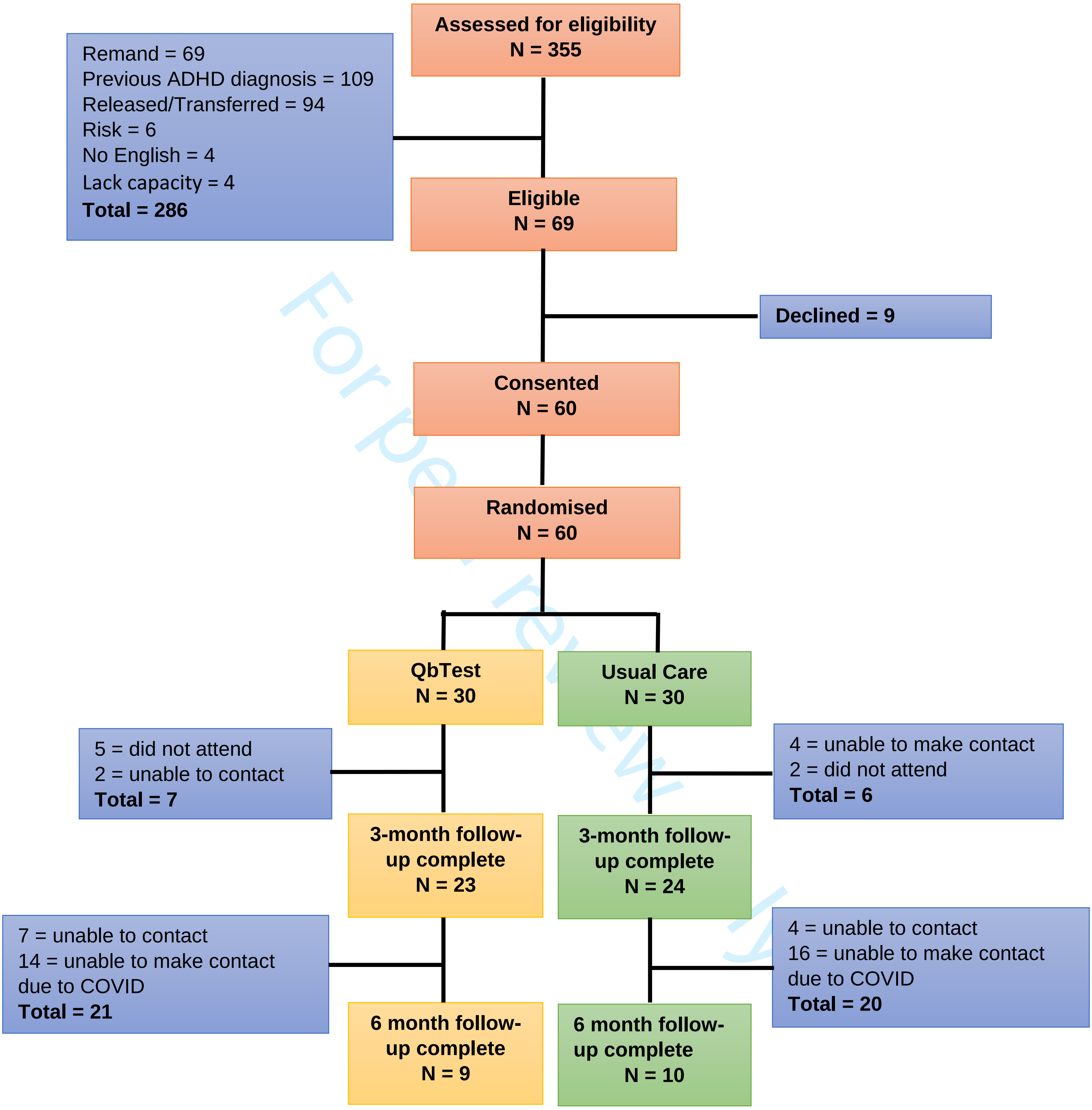

CONSORT 2010 checklist of information to include when reporting a pilot or feasibility trial*

Section/Topic	Item No	Checklist item	Reported on page No
**Title and abstract**			
	1a	Identification as a pilot or feasibility randomised trial in the title	1
	1b	Structured summary of pilot trial design, methods, results, and conclusions (for specific guidance see CONSORT abstract extension for pilot trials)	2-3
**Introduction**			
Background and objectives	2a	Scientific background and explanation of rationale for future definitive trial, and reasons for randomised pilot trial	4-5
	2b	Specific objectives or research questions for pilot trial	5
**Methods**			
Trial design	3a	Description of pilot trial design (such as parallel, factorial) including allocation ratio	5-6
	3b	Important changes to methods after pilot trial commencement (such as eligibility criteria), with reasons	NA
Participants	4a	Eligibility criteria for participants	6
	4b	Settings and locations where the data were collected	6
	4c	How participants were identified and consented	6
Interventions	5	The interventions for each group with sufficient details to allow replication, including how and when they were actually administered	6-7
Outcomes	6a	Completely defined prespecified assessments or measurements to address each pilot trial objective specified in 2b, including how and when they were assessed	7-10
	6b	Any changes to pilot trial assessments or measurements after the pilot trial commenced, with reasons	NA
	6c	If applicable, prespecified criteria used to judge whether, or how, to proceed with future definitive trial	NA
Sample size	7a	Rationale for numbers in the pilot trial	6
	7b	When applicable, explanation of any interim analyses and stopping guidelines	NA
Randomisation:			
Sequence generation	8a	Method used to generate the random allocation sequence	7
	8b	Type of randomisation(s); details of any restriction (such as blocking and block size)	7
Allocation concealment mechanism	9	Mechanism used to implement the random allocation sequence (such as sequentially numbered containers), describing any steps taken to conceal the sequence until interventions were assigned	7

For peer review only

Implementation	10	Who generated the random allocation sequence, who enrolled participants, and who assigned participants to interventions	7
Blinding	11a	If done, who was blinded after assignment to interventions (for example, participants, care providers, those assessing outcomes) and how	7
	11b	If relevant, description of the similarity of interventions	NA
Statistical methods	12	Methods used to address each pilot trial objective whether qualitative or quantitative	10
**Results**			
Participant flow (a diagram is strongly recommended)	13a	For each group, the numbers of participants who were approached and/or assessed for eligibility, randomly assigned, received intended treatment, and were assessed for each objective	6, 11-13
	13b	For each group, losses and exclusions after randomisation, together with reasons	6
Recruitment	14a	Dates defining the periods of recruitment and follow-up	5
	14b	Why the pilot trial ended or was stopped	5-6
Baseline data	15	A table showing baseline demographic and clinical characteristics for each group	Table 1 (p11) and Table 2 (13-14)
Numbers analysed	16	For each objective, number of participants (denominator) included in each analysis. If relevant, these numbers should be by randomised group	NA
Outcomes and estimation	17	For each objective, results including expressions of uncertainty (such as 95% confidence interval) for any estimates. If relevant, these results should be by randomised group	NA
Ancillary analyses	18	Results of any other analyses performed that could be used to inform the future definitive trial	NA
Harms	19	All important harms or unintended effects in each group (for specific guidance see CONSORT for harms)	12
	19a	If relevant, other important unintended consequences	NA
**Discussion**			
Limitations	20	Pilot trial limitations, addressing sources of potential bias and remaining uncertainty about feasibility	17-18
Generalisability	21	Generalisability (applicability) of pilot trial methods and findings to future definitive trial and other studies	17-18
Interpretation	22	Interpretation consistent with pilot trial objectives and findings, balancing potential benefits and harms, and considering other relevant evidence	17-18
	22a	Implications for progression from pilot to future definitive trial, including any proposed amendments	18
**Other information**			
Registration	23	Registration number for pilot trial and name of trial registry	3
Protocol	24	Where the pilot trial protocol can be accessed, if available	10 and 23
Funding	25	Sources of funding and other support (such as supply of drugs), role of funders	19

For peer review only

	26	Ethical approval or approval by research review committee, confirmed with reference number	20

Citation: Eldridge SM, Chan CL, Campbell MJ, Bond CM, Hopewell S, Thabane L, et al. CONSORT 2010 statement: extension to randomised pilot and feasibility trials. BMJ. 2016;355.
*We strongly recommend reading this statement in conjunction with the CONSORT 2010, extension to randomised pilot and feasibility trials, Explanation and Elaboration for important clarifications on all the items. If relevant, we also recommend reading CONSORT extensions for cluster randomised trials, non-inferiority and equivalence trials, non-pharmacological treatments, herbal interventions, and pragmatic trials. Additional extensions are forthcoming: for those and for up to date references relevant to this checklist, see www.consort-statement.org.

For peer review only
